# Incidence and Temporal Trend of Antituberculosis Drug-Induced Liver Injury: A Systematic Review and Meta-Analysis

**DOI:** 10.1155/2022/8266878

**Published:** 2022-10-04

**Authors:** Nannan Wang, Xinyu Chen, Zhuolu Hao, Jia Guo, Xuwen Wang, Xijing Zhu, Honggang Yi, Qingliang Wang, Shaowen Tang

**Affiliations:** ^1^Department of Epidemiology and Biostatistics, School of Public Health, Nanjing Medical University, Nanjing, China; ^2^School of Pharmacy, Nanjing Medical University, Nanjing, China; ^3^First School of Clinical Medicine, Nanjing Medical University, Nanjing, China; ^4^Department of Medical Affairs, Qilu Hospital of Shandong University, Jinan, China

## Abstract

**Methods:**

The Preferred Reporting Items for Systematic Reviews and Meta-Analyses standards were followed, and the protocol was registered in PROSPERO (CRD42020200077). Five electronic databases were searched to identify eligible studies published between 1990 and 2022. Search terms included anti-TB treatment and drug-induced liver injury. Studies that reported the incidence of ATLI or provided sufficient data to calculate the incidence of ATLI were included, and duplicate studies were excluded. Meta-analysis was conducted on the basis of logit-transformed metrics for the incidence of ATLI with 95% confidence intervals (CIs), followed by a predefined subgroup meta-analysis. Temporal trend analyses were performed to describe the change in pooled incidence over time. A random effects metaregression was conducted to explore the source of heterogeneity. All statistical analyses were carried out using *R* 4.0.1.

**Results:**

A total of 160 studies from 156 records with 116147 patients were included in the meta-analysis. Based on the random effects model, the pooled incidence of ATLI was 11.50% (95% CI: 10.10%–12.97%) and showed an upward trend over time (*P* < 0.001). Patients who received first-line anti-TB drugs, patients in South America, and patients with hepatitis B and C virus coinfection had a higher incidence of ATLI (13.66%, 18.16%, and 39.19%, respectively). Sensitivity analyses also confirmed this robust incidence after the exclusion of some studies. The metaregression showed that different anti-TB regimens and geographical regions were important explanatory factors of the heterogeneity between studies.

**Conclusions:**

The present systematic review provided a basis for estimating the incidence of ATLI worldwide, which varied among patients with different anti-TB regimens in different geographical regions and with different coinfections and had an upward trend. Regular liver function monitoring is imperative for patient safety during the anti-TB treatment course.

## 1. Introduction

Tuberculosis (TB), an ancient disease caused by *Mycobacterium tuberculosis*, is one of the top 10 causes of death worldwide and the leading cause of death from a single infectious agent, ranking above HIV/AIDS [1]. Globally, in 2020, an estimated 9.9 million (range 8.9–11.0 million) people contracted TB, and most TB cases were in the regions of Southeast Asia (43%), Africa (25%), and the Western Pacific (18%), with smaller shares in the Eastern Mediterranean (8.3%), the Americas (3.0%) and Europe (2.3%) [2]. Drug treatment is the only effective treatment method for TB, and if applied early in the disease course, these regimens can effectively stop transmission and prevent the disease from spreading [3]. Of the approved drugs, isoniazid (INH), rifampin (RIF), ethambutol (EMB), and pyrazinamide (PZA) are considered first-line anti-TB drugs and form the core of standard treatment regimens [4]. Although these regimens are effective in treating active TB, they are associated with many adverse drug reactions (ADRs) that pose a significant challenge to the completion of treatment [5]. One of the most common ADRs is drug-induced liver injury (DILI), namely, anti-TB DILI (ATLI), which hampers patient adherence to therapy and could negatively impact the treatment outcome of patients [6]. ATLI can be fatal when it is not recognized early and when therapy is not interrupted in time [7].

The incidence of ATLI varies widely depending upon the characteristics of the particular cohort, drug regimens involved, a threshold used to define DILI, monitoring, and reporting practices [8]. Based on previous studies, ATLI has been reported in 5%–28% of people treated with anti-TB drugs [9]. However, among patients with TB, factors including age over 35 years, female sex, elevated pretreatment liver function tests, malnutrition, human immunodeficiency virus (HIV) or hepatitis B virus (HBV) coinfection, and genetic factors increase the risk of ATLI [10–13]. For example, among TB/HIV-coinfected patients, 37% developed liver injury after initiation of anti-TB treatment [14]. Patients with HBV infection also showed a higher frequency of ATLI (14.0%) [13]. Even with standard anti-TB treatment, there are differences in the incidence of ATLI in different regions. Among 55 Indonesian adult TB patients who received standard TB treatment, there were 25 patients (45%) with ATLI [15]. However, in a population-based prospective study with 4304 TB patients receiving a directly observed treatment strategy (DOTS) in China, only 106 patients developed ATLI, with a cumulative incidence of 2.55% [16]. Another anti-TB treatment cohort with 2053 TB patients from China showed that 290 (14.1%) developed ATLI [17]. Additionally, the definition of DILI varied among different studies, such as the international consensus case definition of DILI [18] or the definition from the American Thoracic Society (ATS) [19].

The incidence of ATLI can be used to estimate the magnitude of the harm to the population and the disease burden of the population, and it is also the basis for conducting epidemiological studies and exploring the risk factors for ATLI, especially the calculation of sample size [20]. The incidence of ATLI varies from region to region, and global evidence of these estimates on ATLI is rare. Furthermore, insight into the temporal trend and associated factors will help researchers and policy-makers prepare the clinical infrastructure and healthcare resources needed to mitigate the burden of ATLI. Hence, there is a need for robust aggregation of data on the incidence of ATLI and its temporal trends and identification of potential risk factors for the prevention and control of ATLI. In this regard, the present systematic review aimed to estimate the incidence of ATLI and its temporal trend and provide a basis for developing strategies to reduce the burden of ATLI worldwide.

## 2. Methods

This systematic review and meta-analysis were conducted according to the Preferred Reporting Items for Systematic Reviews and Meta-analyses (PRISMA) statement [21]. This study has been registered on PROSPERO (https://www.crd.york.ac.uk/PROSPERO), and the registration number is CRD42020200077.

### 2.1. Literature Search and Eligibility Criteria

Longitudinal studies reporting the incidence of ATLI in patients with anti-TB treatment were systematically searched in electronic databases, including PubMed, MEDLINE, EMBASE, ClinicalTrials.gov, and the Cochrane Library (Cochrane Central Register of Controlled Trials, CENTRAL), from Jan 1, 1990, to Apr 25, 2022. The search terms fell into two categories: anti-TB treatment and drug-induced liver injury. The computer-based searches combined free terms, MeSH terms, and keywords related to anti-TB treatment and adverse drug reactions with a focus on liver injury. Detailed retrieval strategies for each database are listed in the Supplementary Material (Supplemental Table 1).

All the records identified from the databases mentioned above were imported into EndNote X9.1 (Thomson Reuters, New York, NY), and duplicate records were deleted. Two reviewers independently screened each record by titles, keywords, and abstracts against the eligibility criteria. Full texts were referred to when information in the records was inadequate for evaluation. Any disagreement between the two groups of reviewers was resolved by an additional reviewer.

Studies were included if they met all of the following criteria: (1) study patients had any type of tuberculosis infection caused by *Mycobacterium tuberculosis*; (2) study patients were treated with specific anti-TB drugs; (3) studies reported the incidence of ATLI or provided sufficient data to calculate the incidence of ATLI; (4) data were collected in a prospective manner with a longitudinal study design, including case series, retrospective or prospective cohort studies, or randomized controlled trials; (5) studies were published in English. Additionally, in the case of multiple publications, the study with the most up-to-date or comprehensive information was included. Duplicate studies were excluded from the present study.

### 2.2. Data Extraction

Data extraction was conducted by two independent reviewers with a standardized predesigned data collection form. Data were abstracted, where available, on the study, publication date, geographical location, sample size, number of participants lost to follow-up, age and sex, anti-TB regimens used, diagnostic criteria of ATLI, causality assessment of ATLI, and usage of hepatoprotective drugs. All data were directly taken from the included studies, and no further information was obtained by consulting the authors.

### 2.3. Defining Variables

DILI is caused by medications, herbal and dietary supplements, or other xenobiotics that result in abnormalities in liver tests or in hepatic dysfunction that cannot be explained by other causes [22]. Anti-TB drugs are medicines used to treat TB infection caused by *Mycobacterium tuberculosis* and are available only with a physician's prescription and come in tablet, capsule, liquid, and injectable forms [23]. Hepatoprotective drugs, such as Hu Gan Pian, silymarin, inosine, glucurolactone, and glycyrrhizin, were used to prevent or reduce the occurrence of ATLI [24]. Geographical regions were divided into different groups according to their geographic location in the studies: Africa, Asia, Europe, North America, and South America [25].

### 2.4. Risk of Bias Assessment

The risk of bias in individual studies was assessed by two reviewers using the modified Quality in Prognosis Studies (QUIPS) [26] tool. This tool assesses six domains: study participation, study attrition, prognostic factor measurement, outcome measurement, study confounding, and statistical analysis and reporting. In this tool, the study confounding, statistical analysis, and reporting domains were dropped from the present study, and the item of prognostic factor measurement was modified to the causality assessment of ATLI, which was critical in the judgment of ADRs. Therefore, four domains were included, namely, (1) study participation: The study sample adequately represents the population of interest; (2) study attrition: The study data available (i.e., participants not lost to follow-up); adequately represent the study sample; (3) outcome measurement: ATLI is measured using a clear definition for all participants; (4) Causality assessment: Various types of tools or assessment methods are used to assess the causal relationship between anti-TB drugs and liver injury and are employed to assess the quality of each of the included studies. Each domain was judged carefully by the reviewers and rated as having a high (score of 0), moderate (unclear) (score of 1), or low risk (score of 2) of bias considering the item prompts. Furthermore, each study was then assigned an overall grade of high (score of 0–2), moderate (unclear) (score of 3–5), or low risk (score of 6–8) of bias based on the total score of the four domains.

### 2.5. Statistical Analysis

All statistical analyses were carried out using *R* 4.0.1 (Bell Laboratories, Inc., Madison, WI, USA), and all *P* values were two-tailed. The statistical analysis strategies referred to previous literature [27]. Heterogeneity was assessed via the *Q* test and *I*^2^ statistics. *P* < 0.10 or *I*^2^ >50% indicated substantial heterogeneity [28]. The pooled estimates together with the 95% CIs of the incidence of ATLI were obtained using a DerSimonian-Laird random effects model to accommodate heterogeneity across all included studies. To normalize the distribution of incidence, an arcsine transformation for cumulative incidences was implemented. Publication bias was examined using Egger's test, and the results were considered to have a probable publication bias when *P* < 0.10 [29]. Furthermore, subgroup analysis was performed by sex, diagnostic criteria of ATLI, usage of hepatoprotective drugs, anti-TB regimen, causality assessment, geographical region, coinfection, risk of bias, and type of study to explore the related factors of liver injury and the possible sources of heterogeneity. Moreover, a *Q* test for heterogeneity was used to compare the incidence across subgroups, and 0.05 was defined as the threshold of the *P* value for statistical significance. When the incidence was reported for a multiyear period, the midpoint of the time interval was regarded as the year of the study. A line chart was created to graphically demonstrate the temporal trend of the ATLI incidence, and the trend test of time was carried out using the multivariate Mann-Kendall trend test. Sensitivity analyses were also conducted by omitting studies with a high risk of bias and those without descriptions of ATLI diagnostic criteria, anti-TB drugs, patient age information, patient sex information, and recruitment period. In addition, a multivariate metaregression was conducted to explore the potential source of heterogeneity, and multicategorical variables were defined as dummy variables.

## 3. Results

### 3.1. General Information about the Included Studies

**Figure 1:**
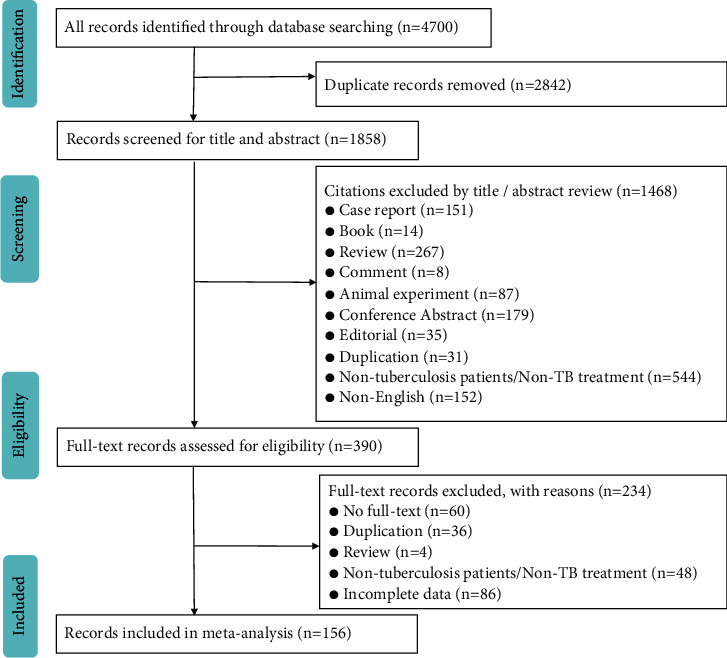
Flow diagram of study inclusion.

**Figure 2:**
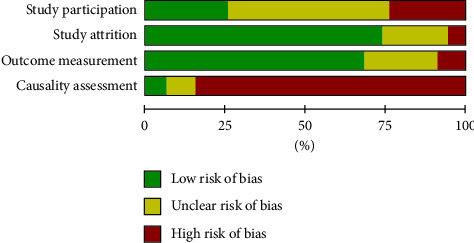
Risk of bias for all the included studies.

A total of 4700 records were identified from five electronic databases, and 160 studies from 156 records with 116147 patients were finally included in the meta-analysis. The flow chart of the included and excluded records is shown in Figure 1. The characteristics of the 160 studies are described in the Supplementary Material (Supplemental Table 2, with the list of all references of the included studies). A summary of the methodological quality of the included studies is illustrated in Supplemental Table 3 and Figure 2. Overall, most of the studies demonstrated a moderate methodology, with a score of 4.6.

### 3.2. Overall Incidence

**Figure 3:**
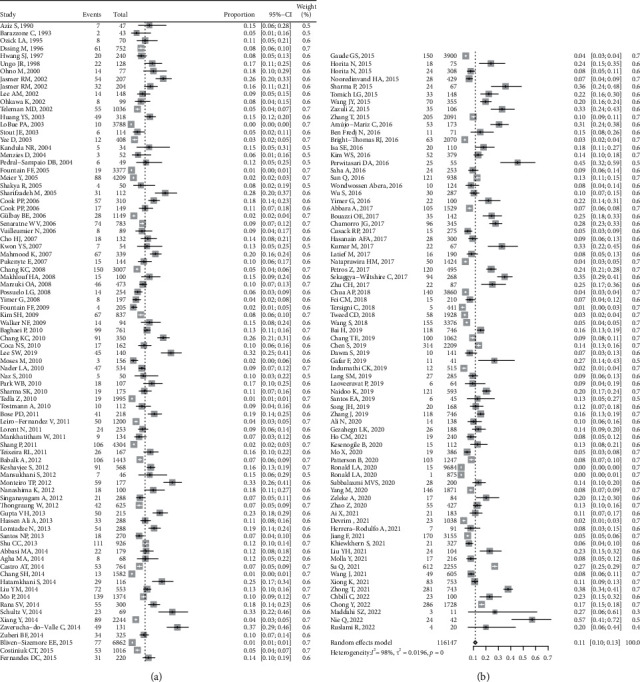
Forrest plot of pooled cumulative incidence of ATLI (random effects model) (ATLI, antituberculosis drug-induced liver injury).

As the forest plot (Figure 3) shows, the pooled cumulative incidence of ATLI was 11.50% (95% CI: 10.10%–12.97%, *I*^2^ = 98.2%) from 1990 to 2022 based on the random effects model. However, Egger's test indicated potential publication bias (*P* < 0.10).

### 3.3. Subgroup Analysis

The results of the subgroup analysis are summarized in Table 1. Patients with first-line anti-TB drugs, patients in South America, and patients with HBV + hepatitis C virus (HCV) coinfection had a higher incidence of ATLI than other patients, with incidences of 13.66% (95% CI: 11.56%–15.89%), 18.16% (95% CI: 12.56%–24.55%), and 39.19% (95% CI: 0.04%–94.45%), respectively. No significant differences were found among different sex, diagnostic criteria of ATLI, usage of hepatoprotective drugs, causality assessment, different risks of bias, and types of study (Table 1). The pooled incidence of ATLI varied across countries, with 36 countries reporting data, and ranged from 1.13% (95% CI: 0.36%–2.33%) in Italy to 35.07% (95% CI: 29.46%–40.90%) in Uganda (Supplemental Table 4).

### 3.4. Temporal Trend

**Figure 4:**
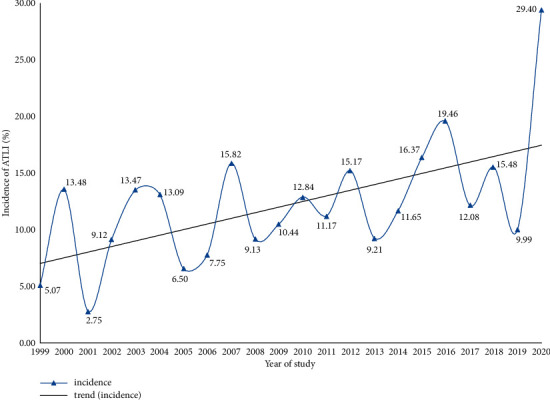
Temporal trend of the pooled incidence of ATLI worldwide (ATLI, antituberculosis drug-induced liver injury).

The pooled incidence of ATLI varied by year, with a lower incidence in 1992 (2.44%, 95% CI: 1.63%–3.41%) and 2001 (2.75%, 95% CI: 0.07%–9.11%) and a higher incidence in 2016 (19.46%, 95% CI: 13.82%–25.79%) and 2020 (29.40%, 95% CI: 14.93%–46.40%). An upward trend was observed in the pooled incidence of ATLI from 1999 to 2020 (*Z* = 5.236, *P* < 0.001) (Figure 4).

### 3.5. Sensitivity Analyses

After excluding studies with a high risk of bias (*n* = 31) and those without a description of ATLI diagnostic criteria (*n* = 18), anti-TB drugs (*n* = 19), patient age information (*n* = 28), patient sex information (*n* = 17), and patient recruitment period (*n* = 12), the pooled incidences of ATLI were 11.52% (95% CI: 9.98%–13.15%), 11.82% (95% CI: 10.44%–13.27%), 11.41% (95% CI: 9.91%–13.01%), 11.84% (95% CI: 10.32%–13.45%), 11.60% (95% CI: 10.18%–13.09%), and 11.34% (95% CI: 9.90%–12.87%), respectively, and the incidence of ATLI remained stable (Table 2).

### 3.6. Metaregression

Table 3 summarizes the meta-analysis results, and the multivariate metaregression model showed that different anti-TB regimens and geographical regions might be significant sources of heterogeneity (*P* < 0.05), which together explained 21.04% of the total variance between studies.

## 4. Discussion

To the best of our knowledge, this is the first systematic review and meta-analysis on the incidence of ATLI in the world, and the pooled incidence of ATLI in the past 30 years was robust. Furthermore, patients who received first-line anti-TB drugs, patients in South America, and patients with HBV + HCV coinfection had a higher incidence of ATLI than other patients, while sex and the hepatoprotective effect did not display a significant difference in the pooled incidence of ATLI. Although data from only 36 countries were available in the present study, the statistical map also suggested differences in the incidence of ATLI between countries. This study has positive significance for understanding the current global status of ATLI, assessing its disease burden, and implementing ATLI prevention and control measures.

Identification of the risk factors for ATLI is essential to prevent ATLI and improve patient outcomes. Conclusions from different studies were inconsistent regarding whether sex was related to the occurrence of ATLI, namely, whether male sex was a risk factor [30] or female sex was a risk factor [31] or was associated with the severity of ATLI [32]. However, as early as 2006, the official statement from ATS showed that there was currently no clear evidence to point to an overall sex-related difference in the incidence of hepatotoxicity [19], and our present study further confirmed this result. Additionally, whether the prophylactic use of hepatoprotective drugs affects the occurrence of ATLI is another controversial issue. Different studies found that the use of hepatoprotective drugs significantly decreased the number of ATLI cases [33], no preventive effect of hepatoprotective drugs was observed [24], or a potential risk of liver injury was caused by hepatoprotective drugs [34]. Of the 160 studies included in the present study, although only eight studies included prophylactic hepatoprotective drugs, the results of the meta-analysis indicated a similar pooled incidence of ATLI in studies with or without hepatoprotective drugs. Compared with other risk factors, the pooled incidences of ATLI in TB patients with hepatitis or HIV were higher (Table 1), with the highest incidence in patients with HBV + HCV coinfection. A previous study revealed that HBV infection was only associated with transient liver function impairment, while HCV is the real independent risk factor for ATLI [35]. The present systematic review included studies from five continents (Asia, Africa, Europe, South America, and North America) and 36 countries. In these countries, the highest pooled incidence of ATLI was in Uganda, while the lowest was in Italy. This difference was associated with multiple factors, such as race, social and economic status, geographical position, the diagnostic criteria that researchers adopted, prevalence, and preventive treatment of viral hepatitis [36].

Because the mechanism underlying DILI is not well understood, there is still no single biomarker or diagnostic tool to unequivocally diagnose DILI [37]. Many parameters with different thresholds are also currently used in clinical practice, such as the international consensus criteria (alanine aminotransferase (ALT), conjugated bilirubin, or alkaline phosphatase (ALP) >2 × the upper limit of normal (ULN)) [18], the DILI Network criteria (ALT or aspartate aminotransferase (AST) >5 × ULN and/or ALP >2 × ULN [38]), the official American Thoracic Society statement (ALT or AST >3 × ULN with symptoms or 5 × ULN of ALT or AST without symptoms [19]), and the international DILI Expert Working Group consensus statement (ALT ≥5 × ULN or ALT ≥3 × ULN with total bilirubin >2 × ULN [39]. In addition to these common diagnostic thresholds, this study also found other thresholds, such as ALT >1 × ULN [14], 1.25 × ULN [40], or 2.5 × ULN [41], which was also the key to the differences in the ATLI incidence between studies. The results from the present study also indicated that the diagnostic threshold ranged from ALT >2 × the ULN to >5 × the ULN, and its corresponding pooled incidence also decreased from 15.00% to 9.81% (Table 1). Additionally, because there are no specific tests to confirm the diagnosis of DILI, a causality assessment must be employed to establish a definitive link between drug intake and liver injury [39]. Many systems or tools have been developed for a more standardized, less subjective assessment of causality [42]. Although each has its drawbacks, causality assessment has become a common routine procedure in pharmacovigilance [43]. However, of the 160 included studies in this systematic review, only 24 had causality assessments. Perhaps many judgments of ATLI were still based on the doctor's personal experience [42] or only on the results of liver function tests.

In the present study, the pooled incidence curve for ATLI showed a clear upward trend worldwide, which is consistent with other research findings [44, 45]. In recent years, with the extensive use of drugs and the accelerated research of novel drugs, the different types of clinical drugs have been continuously increasing, and the incidence of DILI has been rising year by year [46], and ATLI is no exception. In addition to the common first- and second-line anti-TB drugs, there are third-line drugs that are used to treat drug-resistant TB but typically have less activity, more adverse reactions, and less evidence supporting their use than first- and second-line drugs [47]. Furthermore, the global burden of multidrug-resistant TB has recently increased by an annual rate of more than 20% [48]. Additionally, DILI has attracted increasing interest among researchers in recent years. The definition, incidence rate or clinical characteristics, etiology or pathogenesis, identification of main drugs, and causality assessment were the knowledge base for DILI research [49], and the pathogenesis, clinical manifestations, diagnosis, and risk factors of DILI were the most prominent research hotspots [50]. All of these may be responsible for the increasing incidence of ATLI in the world.

There are several potential limitations of the present systematic review. First, 60.6% of the included studies were of moderate methodological quality. However, study quality did not appear to be the main source of heterogeneity according to the subgroup analyses and metaregression. Sensitivity analyses that were carried out by omitting studies with a high risk of bias produced robust results as well. Second, possible publication bias should be considered when evaluating the results. Limiting the study to English-language articles may have potentially led to a language bias. Third, the heterogeneity statistic *I*^2^ was very high in the present study. After metaregression was performed in this systematic review, only 21.04% of the total variance could be explained. Indeed, there are arguments against pooling estimates in the presence of extensive heterogeneity. However, this systematic review included studies attempting to measure the same outcome, and a high *I*^2^ may be driven by the substantial number of large, precise studies [51]; therefore, the average estimates remain useful.

## 5. Conclusion

In conclusion, the present systematic review provided a basis for estimating the incidence of ATLI worldwide, which varied among patients with different anti-TB regimens, in different geographical regions, and with different coinfections and had an upward trend. Risk assessment and regular liver function monitoring are imperative for patient safety during the anti-TB treatment course.

## Figures and Tables

**Table 1:** The results of overall and subgroup analyses by different variables.

Study-level variables	Number of studies	Sample size	Pooled incidence (%)	95% CI (%)	*I* ^2^ (%)	*P* value
Overall	160	116147	11.50	10.10–12.97	98.2	—

Sex						0.0663
Male	62	22591	11.27	9.21–13.51	96.0	
Female	62	17427	11.29	9.15–13.62	94.8	

Diagnostic criteria						0.1772
ALT>2ULN	28	19790	15.00	11.04–19.44	98.4	
ALT>3ULN	83	51761	11.24	9.54–13.06	97.4	
ALT>5ULN	20	16419	9.81	7.40–12.51	95.9	
Other^a^	29	28177	10.15	6.95–13.88	98.7	

Hepatoprotective drugs						0.7906
Yes	8	8223	11.86	9.29–14.70	91.6	
No	152	107924	11.45	10.00–12.98	98.2	

Anti-TB regimen						<0.0001
HRZES	25	28617	9.85	7.52–12.45	97.8	
HRZE	69	34624	13.66	11.56–15.89	96.9	
HRZ	19	6609	12.22	9.21–15.58	93.2	
H	12	28987	2.87	1.75–4.24	96.8	
Other^b^	16	6089	11.04	6.64–16.38	96.9	

Causality assessment						0.2516
Yes	24	29711	10.05	7.81–12.53	97.6	
No	136	86436	11.78	10.11–13.56	98.3	

Geographical regions						<0.0001
Asia	82	60794	12.65	10.96–14.44	97.5	
Africa	17	5379	12.71	7.79–18.61	96.8	
Europe	22	16620	6.75	4.97–8.79	95.1	
South America	14	2744	18.16	12.56–24.55	93.9	
North America	25	30610	7.98	5.35–11.08	98.6	

Coinfection						<0.0001
HBV	20	10665	20.07	14.41–26.41	80.3	
HCV	4	1279	25.05	18.03–32.80	0	
HIV	30	17620	21.49	16.09–27.45	92.3	
HBV + HCV	2	932	39.19	0.04–94.45	82.5	
HBV + HIV	2	416	29.08	17.94–41.67	0	

Risk of bias						0.8376
High	31	17313	11.43	8.03–15.34	98.0	
Moderate	97	75832	11.27	9.47–15.50	98.4	
Low	32	23002	12.33	9.47–13.20	97.7	

Type of study						0.6617
Cohort study	107	79131	10.83	9.15–12.63	98.3	
Nested case-control	15	19155	12.99	8.62–18.10	98.7	
RCT	16	6751	13.16	7.85–19.60	97.9	
Case series	22	11110	12.32	9.56–15.36	94.6	

CI, confidence interval; ALT, alanine aminotransferase; ULN, upper normal value limit; *H*, isoniazid; *R*, rifampin; *E*, ethambutol; *Z*, pyrazinamide; *S*, streptomycin; HIV, human immunodeficiency virus; HBV, hepatitis B virus; HCV, hepatitis C virus; RCT, randomized controlled trial; ^a^including ALT >1ULN, ALT >1.25ULN, ALT >2.5ULN, or AST >2ULN. ^b^including RZ, HR, HRE, *R* or HR + E/S.

**Table 2:** Sensitivity analysis of pooled ATLI incidence after the exclusion of some studies.

Reason for exclusion	Number of excluded studies	Pooled incidence (%)	95% CI (%)	*I* ^2^ (%)
High risk of bias	31	11.52	9.98–13.15	98.3
Without ATLI diagnostic criteria	18	11.82	10.44–13.27	97.7
Without descriptions of anti-TB drugs	19	11.41	9.91–13.01	98.3
Without patient age information	28	11.84	10.32–13.45	97.9
Without patient sex information	17	11.60	10.18–13.09	97.9
Without patient recruitment period	12	11.34	9.90–12.87	98.3

ATLI, antituberculosis drug-induced liver injury; TB, tuberculosis; CI, confidence interval.

**Table 3:** Results of the metaregression in the pooled ATLI incidence analysis.

Study-level variables	*β* coefficient	Standard error	95% CI	*P* value
Diagnostic criteria				
ALT>2 ULN	Reference			
ALT>3 ULN	−0.0319	0.0297	−0.0900 to 0.0263	0.2826
ALT>5 ULN	−0.0383	0.0404	−0.1175 to 0.0409	0.3431
Others	−0.0060	0.0393	−0.0831 to 0.0710	0.8778

Hepatoprotective drugs	−0.0391	−0.0527	−0.1424 to 0.0641	0.4578

Anti-TB regimen				
H	Reference			
RHZES	0.1256	0.0549	0.0180 to 0.2332	0.0222
RHZE	0.1985	0.0471	0.1062 to 0.2908	<0.0001
RHZ	0.1365	0.0545	0.0297 to 0.2434	0.0123
Others	0.1691	0.0479	0.0751 to 0.2630	0.0004

Causality assessment	0.0451	0.0358	−0.0251 to 0.1153	0.2081

Geographical regions				
North America	Reference			
Africa	0.0287	0.0482	−0.0657 to 0.1232	0.5511
South America	0.1379	0.0511	0.0377 to 0.2381	0.0070
Europe	−0.0559	0.0426	−0.1394 to 0.0275	0.1889
Asia	0.0360	0.0376	−0.0378 to 0.1097	0.3389

Coinfection				
HBV	Reference			
HIV	−0.0705	0.0534	−0.1752 to 0.0342	0.1869
Others	−0.0510	0.0441	−0.1375 to 0.0355	0.2479

Risk of bias				
High	Reference			
Moderate	−0.0328	0.0279	−0.0875 to 0.0219	0.2392
Low	0.0023	0.0373	−0.0709 to 0.0754	0.9517

Type of study				
Case series	Reference			
RCT	0.0161	0.0329	−0.0764 to 0.1085	0.7334
Nested case-control	0.0372	0.0459	−0.0527 to 0.1271	0.4173
Cohort study	−0.0023	0.0472	−0.0668 to 0.0623	0.9454

ATLI, antituberculosis drug-induced liver injury; TB, tuberculosis; CI, confidence interval; ALT, alanine aminotransferase; ULN, upper normal value limit; *H*, isoniazid; *R*, rifampin; *E*, ethambutol; *Z*, pyrazinamide; *S*, streptomycin; RCT, randomized controlled trial.

## Data Availability

The data supporting the findings of this study are included within the main manuscript and the supplemental files.

## Abbreviations

ADR: Adverse drug reaction
ALP: Alkaline phosphatase
ALT: Alanine aminotransferase
ATLI: Antituberculosis drug-induced liver injury
DILI: Drug-induced liver injury
DOTS: Directly observed treatment strategy
EMB: Ethambutol
HBV: Hepatitis B virus
HIV: Human immunodeficiency virus
INH: Isoniazid
PRISMA: Preferred reporting items for systematic reviews and meta-analyses
PZA: Pyrazinamide
QUIPS: Quality in prognosis studies
RIF: Rifampin
TB: Tuberculosis
ULN: Upper limit of normal.

## Supplementary Materials

Supplementary Materials Supplementary materials are given in the .docx file format. Supplemental Table 1: search strategies and results. Supplemental Table 2: general information of the included studies. Supplemental Table 3: summary of risk of bias assessment. Supplemental Table 4: the pooled incidence of ATLI in different countries.
