# Supplementary material for: Incidence and Temporal Trend of Antituberculosis Drug-Induced Liver Injury: A Systematic Review and Meta-Analysis
Source: J Trop Med. 2022 Oct 4;2022:8266878. doi: 10.1155/2022/8266878 (PMC9553535; doi:10.1155/2022/8266878)
Supplement: Supplementary Materials — are given in the .docx file format. Supplemental Table 1: search strategies and results. Supplemental Table 2: general information of the included studies. Supplemental Table 3: summary of risk of bias assessment. Supplemental Table 4: the pooled incidence of ATLI in different countries. [file 8266878.f1.docx]

Supplemental Table 1. Search strategies and results

PubMed

| #9 | Search #3 and #8 | 1466 |
| --- | --- | --- |
| #8 | Search #6 or #7 | 35868 |
| #7 | Search "anti-tuberculosis drug-induced liver injury"[Title/Abstract]) OR (" anti-tuberculosis drug-induced liver condition "[Title/Abstract])) OR ("anti-tuberculosis drug-induced liver disease"[Title/Abstract])) OR ("anti-tuberculosis drug-induced liver disorder"[Title/Abstract])) OR ("anti-tuberculosis drug-induced liver failure "[Title/Abstract])) OR (" anti-tuberculosis drug-induced liver damage"[Title/Abstract])) OR ("anti-tuberculosis drug-induced hepatotoxicity "[Title/Abstract])) OR ("anti-tuberculosis drug-induced hepatic injury "[Title/Abstract])) OR ("anti-tuberculosis drug induced liver injury"[Title/Abstract])) OR (anti-tuberculosis drug-induced liver injury) | 288 |
| #6 | Search #4 or #5 | 35860 |
| #5 | Search "drug-induced liver injury"[Title/Abstract] OR "drug-induced liver disease"[Title/Abstract] OR "drug-induced liver failure"[Title/Abstract] OR "drug-induced liver damage"[Title/Abstract] OR "drug-induced hepatotoxicity"[Title/Abstract] OR "drug-induced hepatic injury"[Title/Abstract] OR "drug-induced hepatitis"[Title/Abstract] OR "drug-induced cholestasis"[Title/Abstract] OR "idiosyncratic liver disease"[Title/Abstract] OR "toxic hepatitis"[Title/Abstract] OR "toxic liver disease"[Title/Abstract] | 7711 |
| #4 | Search Drug induced liver injury [MeSH] | 32148 |
| #3 | Search #1 or #2 | 299196 |
| #2 | Search tubercul* | 298812 |
| #1 | Search tuberculosis | 277673 |

MEDLINE

| #7 | Search #6 and #3 | 1422 |
| --- | --- | --- |
| #6 | Search #4 or #5 | 34493 |
| #5 | Search TS=("anti-tuberculosis drug-induced liver injury" OR "anti-tuberculosis drug-induced liver condition" OR "anti-tuberculosis drug-induced liver disease" OR "anti-tuberculosis drug-induced liver disorder" OR "anti-tuberculosis drug-induced liver failure" OR "anti-tuberculosis drug-induced liver damage" OR "anti-tuberculosis drug-induced hepatotoxicity" OR "anti-tuberculosis drug-induced hepatic injury" OR "anti-tuberculosis drug induced liver injury" OR "anti-tuberculosis drug-induced liver injury") | 122 |
| #4 | Search TS=("Drug induced liver injury" OR "drug-induced liver injury" OR "drug-induced liver condition" OR "drug-induced liver disease" OR "drug-induced liver disorder" OR "drug-induced liver failure" OR "drug-induced liver damage" OR "drug-induced hepatotoxicity" OR "drug-induced hepatic injury" OR "drug-induced hepatitis" OR "drug-induced cholestasis" OR "idiosyncratic liver condition" OR "idiosyncratic liver disease" OR "idiosyncratic liver disorder" OR "toxic hepatitis" OR "toxic liver condition" OR "toxic liver disease" OR "toxic liver disorder") | 34493 |
| #3 | Search #1 or #2 | 374509 |
| #2 | Search TS= tubercul* | 374509 |
| #1 | Search TS=Tuberculosis | 341686 |

Embase

| #1 | Search 'tuberculosis'/exp | 284144 |
| --- | --- | --- |
| #2 | Search 'tubercul*':ti,ab | 283817 |
| #3 | Search #1 or #2 | 356322 |
| #4 | Search 'toxic hepatitis'/exp | 16042 |
| #5 | Search 'drug induced liver injury':ab,ti | 5566 |
| #6 | Search 'drug-induced liver injury':ti,ab OR 'drug-induced liver condition':ti,ab OR 'drug-induced liver disease':ti,ab OR 'drug-induced liver disorder':ti,ab OR 'drug-induced liver failure':ti,ab OR 'drug-induced liver damage':ti,ab OR 'drug-induced hepatotoxicity':ti,ab OR 'drug-induced hepatic injury':ti,ab OR 'drug-induced hepatitis':ti,ab OR 'drug-induced cholestasis':ti,ab OR 'idiosyncratic liver condition':ti,ab OR 'idiosyncratic liver disease':ti,ab OR 'idiosyncratic liver disorder':ti,ab OR 'toxic liver condition':ti,ab OR 'toxic liver disease':ti,ab OR 'toxic liver disorder':ti,ab | 8162 |
| #7 | Search 'anti-tuberculosis drug-induced liver condition':ti,ab OR 'anti-tuberculosis drug-induced liver disease':ti,ab OR 'anti-tuberculosis drug-induced liver disorder':ti,ab OR 'anti-tuberculosis drug-induced liver failure':ti,ab OR 'anti-tuberculosis drug-induced liver damage':ti,ab OR 'anti-tuberculosis drug-induced hepatotoxicity':ti,ab OR 'anti-tuberculosis drug-induced hepatic injury':ti,ab OR 'anti-tuberculosis drug induced liver injury':ti,ab OR 'anti-tuberculosis drug-induced liver injury':ti,ab | 161 |
| #8 | Search #4 or #5 or #6 or #7 | 19688 |
| #9 | Search #3 and #8 | 1637 |

Clinical Trials.gov

17 Studies found for: Tuberculosis | Drug induced liver injury

| Terms | Search Results* | Entire Database** |
| --- | --- | --- |
| Synonyms |  |  |
| Tuberculosis | 17studies | 2392studies |
| Tuberculous | 4studies | 131 studies |
| Drug induced liver injury | 17 studies | 215 studies |
| Drug-induced hepatotoxicity | 5 studies | 7studies |
| Drug-induced disorder of liver | -- | 1 study |
| Drug Induced Liver Disease | -- | 7 studies |
| li**ver injury** | 13studies | 650 studies |
| Hepatic injury | 2 studies | 56 studies |
| Injury of liver | -- | 3 studies |
| Injury to liver | -- | 1 study |
| Drug induced | 17 studies | 723 studies |
| injury | 14 studies | 36051 studies |
| wounds | 3studies | 19827 studies |
| Trauma | -- | 10167 studies |
| liver | 15studies | 19635studies |
| Hepar | -- | 7 studies |
| induced | 17studies | 36087studies |
| Induce | -- | 3971 studies |
| Inducible | -- | 421 studies |
| inducing | -- | 1605studies |
| Induction | -- | 10177 studies |
| Drug | 17 studies | 239742studies |
| Medicine | 5 studies | 61583studies |
| Agent | 13 studies | 13911 studies |
| Medications | 1 study | 36730studies |
| Pharmaceutic Preparations | -- | 1 study |
| Pharmaceutical | 1 study | 24587studies |
| Medicinal products | 1 study | 2484studies |
| Pharmacologic Substance | -- | 1 study |
| Pharmacological Substance | -- | 6 studies |

10 Studies found for: Tuberculosis | Completed Studies | Drug induced liver injury

| Terms | Search Results* | Entire Database** |
| --- | --- | --- |
| Synonyms |  |  |
| Tuberculosis | 10 studies | 1228 studies |
| Tuberculous | 1 study | 55 studies |
| Drug induced liver injury | 10 studies | 215studies |
| Drug-induced hepatotoxicity | 2studies | 7 studies |
| Drug Induced Liver Disease | -- | 7 studies |
| Drug-induced disorder of liver | -- | 1 study |
| liver injury | 9studies | 650 studies |
| Hepatic injury | 1 study | 56studies |
| Injury of liver | -- | 3 studies |
| Injury to liver | -- | 1 study |
| Drug induced | 10 studies | 723studies |
| injury | 9 studies | 36051 studies |
| wounds | 1study | 19827studies |
| Trauma | -- | 10167 studies |
| liver | 5 studies | 19635studies |
| Hepar | -- | 47studies |
| induced | 10studies | 36087studies |
| Induce | -- | 3971 studies |
| Inducible | -- | 421 studies |
| inducing | -- | 1605 studies |
| Induction | -- | 10177 studies |
| Drug | 10 studies | 239742 studies |
| Agent | 8 studies | 13911 studies |
| Medications | -- | 36730 studies |
| Medicine | 3studies | 61583 studies |
| Pharmaceutic Preparations | -- | 1 study |
| Pharmaceutical | 1 study | 24587tudies |
| Medicinal products | 1 study | 2484studies |
| Pharmacologic Substance | -- | 1 study |
| Pharmacological Substance | -- | 6 studies |

Cochrane Library - Cochrane Central Register of Controlled Trials

| #14 | Search #3 and #13 | 158 |
| --- | --- | --- |
| #13 | Search #12 or #9 | 1872 |
| #12 | Search #10 or #11 | 28 |
| #11 | Search ('anti-tuberculosis drug-induced liver damage'):ti,ab,kw OR ('anti-tuberculosis drug-induced hepatotoxicity '):ti,ab,kw OR ('anti-tuberculosis drug-induced hepatic injury '):ti,ab,kw OR ('anti-tuberculosis drug induced liver injury'):ti,ab,kw OR ('anti-tuberculosis drug-induced liver injury'):ti,ab,kw | 27 |
| #10 | Search ('anti-tuberculosis drug-induced liver injury'):ti,ab,kw OR ('anti-tuberculosis drug-induced liver condition '):ti,ab,kw OR ('anti-tuberculosis drug-induced liver disease'):ti,ab,kw OR ('anti-tuberculosis drug-induced liver disorder'):ti,ab,kw OR ('anti-tuberculosis drug-induced liver failure'):ti,ab,kw | 27 |
| #9 | Search #4 or #5 or #6 or #7 or #8 | 1872 |
| #8 | Search ('toxic liver disease'):ti,ab,kw OR ('toxic liver disorder'):ti,ab,kw | 546 |
| #7 | Search ('idiosyncratic liver condition'):ti,ab,kw OR ('idiosyncratic liver disease'):ti,ab,kw OR ('idiosyncratic liver disorder'):ti,ab,kw OR ('toxic hepatitis'):ti,ab,kw OR ('toxic liver condition'):ti,ab,kw | 427 |
| #6 | Search ('drug-induced liver damage'):ti,ab,kw OR ('drug-induced hepatotoxicity'):ti,ab,kw OR ('drug-induced hepatic injury'):ti,ab,kw OR ('drug-induced hepatitis'):ti,ab,kw OR ('drug-induced cholestasis'):ti,ab,kw | 540 |
| #5 | Search ('drug induced liver injury'):ti,ab,kw OR ('drug-induced liver condition'):ti,ab,kw OR ('drug-induced liver disease'):ti,ab,kw OR ('drug-induced liver disorder'):ti,ab,kw OR ('drug-induced liver failure'):ti,ab,kw | 1037 |
| #4 | Search ('Drug induced liver injury'):ti,ab,kw | 835 |
| #3 | Search #1 or #2 | 7766 |
| #2 | Search ('tubercul*'):ti,ab,kw | 7765 |
| #1 | Search MeSH descriptor: [Tuberculosis] explode all trees | 2474 |

Supplemental Table 2. General information of the included studies

| Study | Country | Study design | Recruitment period | No. of TB patients | Mean age | Male (%) | Anti-TB drugs | No. of ATLI | Criteria of ATLI |
| --- | --- | --- | --- | --- | --- | --- | --- | --- | --- |
| Chbili 2022[1] | Tunisia | case series | Jan.2015-Jan.2016 | 100 | - | - | HRZE | 23 | ALT>2ULN |
| Chong 2022[2] | China | case series | Jul.2019-Dec.2020 | 1728 | 46.00 | - | HRZES | 286 | ALT>3ULN |
| Maddahi 2022[3] | Iran | RCT | Nov.2019-Jun.2020 | 11 | - | - | HRZE | 3 | Not clear |
| Nie 2022[4] | China | case series | Jul.2019-Jul.2020 | 42 | - | 57.14 | HZE | 24 | Not clear |
| Ruslami2022[5] | Netherlands | case series | Mar.2018-Jan.2020 | 20 | 11.40 | 45.00 | HRZE | 4 | ALT>3ULN |
| Ai 2021[6] | China | RCT | Jun.2018-Mar.2019 | 183 | - | - | HRZE | 21 | Not clear |
| Devrim 2021[7] | Turkey | cohort study | Dec.2009-Aug.2013 | 1038 | - | 44.22 | H | 23 | ALT>3ULN |
| Herrera 2021[8] | Mexico | RCT | - | 91 | 38.60 | 56.04 | HRZE | 7 | ALT>2ULN |
| Jiang 2021[9] | China | cohort study | Jul.2011-Dec.2015 | 3155 | 37.00 | 64.00 | HRZE | 170 | ALT>5ULN |
| Khiewkhern 2021[10] | Thailand | case series | 2016-2018 | 327 | 51.00 | 63.91 | HRZE | 21 | ALT>3ULN |
| Liu 2021[11] | China | case series | Jan.2019-Mar.2021 | 104 | 56.41 | 53.85 | HRZE | 24 | ALT>3ULN |
| Molla 2021[12] | Ethiopia | case series | Dec.2017-Jun.2019 | 216 | 43.00 | 45.37 | HRZE | 17 | ALT>3ULN |
| Su 2021[13] | China | cohort study | Jan.2015-Dec.2019 | 2255 | - | - | HRZE | 612 | ALT>2ULN |
| Wang 2021[14] | China | cohort study | Jan.2011-Dec.2013 | 605 | - | 72.73 | Not clear | 49 | ALT>2ULN |
| Xiong 2021[15] | China | RCT | Oct.2012-Mar.2015 | 753 | - | 76.10 | HRZE | 83 | ALT>3ULN |
| Zhong 2021[16] | China | RCT | 2014-2019 | 743 | - | 65.14 | HRZE | 281 | ALT>3ULN |
| Ali 2020[17] | India | cohort study | Sep.2017-Aug.2019 | 138 | 46.30 | 61.59 | Not clear | 14 | ALT>3ULN |
| Gezahegn 2020[18] | Ethiopia | case series | Aug.2015-Jun.2018 | 188 | - | 56.38 | HRZE | 26 | ALT>3ULN |
| Ho 2020[19] | China | cohort study | Dec.2011-Jul.2017 | 240 | - | 59.58 | Not clear | 19 | ALT>5ULN |
| Kesenogile 2020[20] | Botswana | case series | Jun.2017-Jun.2018 | 112 | - | 35.71 | HRZE | 15 | ALT>3ULN |
| Mo 2020[21] | China | cohort study | Jun.2002-Jun.2018 | 386 | 40.00 | 36.27 | Not clear | 19 | ALT>3ULN |
| Patterson 2020[22] | UK | cohort study | Jan.2015-Dec.2018 | 1247 | 50.53 | 57.42 | Not clear | 103 | ALT>3ULN |
| Ronald 2020[23] | Canada | cohort study | 2003-2007 | 9684 | - | 42.91 | H | 15 | - |
| Ronald 2020[23] | Canada | cohort study | 2003-2007 | 875 | - | 48.11 | R | 1 | - |
| Subbalaxmi 2020[24] | India | case series | - | 200 | 36.00 | 56.50 | HRZE | 28 | ALT>3ULN |
| Yang 2020[25] | China | case series | May.2016-Dec.2018 | 1871 | - | - | HRZES | 146 | ALT>3ULN |
| Zeleke 2020[26] | Ethiopia | case series | Sep.2015-Aug.2018 | 84 | 41.00 | 52.38 | Not clear | 17 | ALT>3ULN |
| Zhao 2020[27] | China | cohort study | Nov. 2011-Sep. 2015 | 427 | 42.45 | 61.36 | HR | 55 | ALT>3ULN |
| Bai 2019[28] | China | RCT | Dec.2016-Apr.2018 | 746 | 42.55 | 59.52 | Not clear | 118 | ALT>3ULN |
| Chang 2019[29] | China | cohort study | 2009-2017 | 1062 | 67.10 | 70.43 | HRZES | 100 | ALT>5ULN |
| Chen 2019[30] | China | nested case-control | Apr.2014- Dec.2016 | 2209 | - | - | HRZES | 314 | ALT>2ULN |
| Dawra 2019[31] | India | cohort study | - | 141 | 34.30 | 51.06 | HRZE | 10 | ALT>3ULN |
| Gafar 2019[32] | Indonesia | cohort study | Sep.2015-Apr.2016 | 41 | - | 63.41 | HRZE | 11 | ALT>3ULN |
| Indumathi 2019[33] | India | RCT | Jan.2007-Dec.2017 | 515 | 5.50 | - | HRZE | 12 | Not clear |
| Lang 2019[34] | Germany | cohort study | 2009-2017 | 285 | - | - | HRZE | 27 | ALT>3ULN |
| Laoveeravat2019[35] | Thailand | cohort study | Jan.2006-Apr.2016 | 64 | - | 70.31 | Not clear | 6 | ALT>3ULN |
| Naidoo 2019[36] | USA | RCT | Jun.2015-Jul.2010 | 593 | - | 49.41 | HRZES | 121 | ALT>5ULN |
| Santos 2019[37] | Brazil | nested case-control | Aug.2012-Sep.2013 | 45 | 42.00 | 66.67 | Not clear | 6 | ALT>2ULN |
| Song 2019[38] | Korea | nested case-control | Jan.2013-Dec.2016 | 168 | 53.40 | 66.07 | HRZE | 20 | ALT>3ULN |
| Zhang 2019[39] | China | cohort study | Dec.2014- Apr.2018 | 746 | - | - | HRZE | 118 | ALT>3ULN |
| Cheah 2018[40] | Malaysia | case series | Jan.2017-Mar.2017 | 210 | - | 73.33 | HRZES | 15 | ALT>3ULN |
| Chua 2018[41] | Singapore | cohort study | Jan.2013- Dec.2014 | 3860 | - | 46.68 | HRZES | 140 | ALT>2ULN |
| Tersigni 2018[42] | Italy | cohort study | Jan.2017- Dec.2016 | 441 | 8.17 | 57.14 | HR | 5 | ALT>3ULN |
| Tweed 2018[43] | UK | RCT | - | 1928 | - | - | HRZES | 58 | ALT>3ULN |
| Wang 2018[44] | China | cohort study | 2010-2016 | 3376 | - | - | HRZE | 155 | ALT>5ULN |
| Abbara 2017[45] | UK | cohort study | Apr.2010-May.2014 | 1529 | - | - | Not clear | 105 | ALT>3ULN |
| Chamorro 2017[46] | Argentina | nested case-control | 2009-2015 | 345 | 29.00 | 62.90 | Not clear | 96 | ALT>3ULN |
| Cusack 2017[47] | Ireland | cohort study | 2009-2014 | 275 | 43.75 | 61.82 | HRZE | 15 | Not clear |
| Hasanain 2017[48] | Egypt | RCT | Oct.2014-May.2016 | 300 | 35.60 | 43.00 | HRZE | 28 | ALT>2ULN |
| Kumar 2017[49] | USA | cohort study | Aug.2014- Oct.2016 | 67 | 30.00 | 43.28 | HRZE | 22 | ALT>3ULN |
| Latief 2017[50] | India | cohort study | - | 190 | - | 64.21 | HRZE | 16 | ALT>3ULN |
| Nataprawira 2017[51] | Indonesia | case series | Oct.2010- Oct.2015 | 1424 | - | - | HRZE | 50 | Not clear |
| Petros 2017[52] | Sweden | nested case-control | Jun.2007-Jun.2012 | 495 | - | - | HRZE | 120 | ALT>3ULN |
| Sekaggya 2017[53] | Uganda | cohort study | May.2013-Nov.2015 | 268 | 34.00 | 57.84 | HRZE | 94 | ALT>1ULN |
| Zhu 2017[54] | China | cohort study | Jun.2007-Jun.2012 | 87 | 44.38 | 78.16 | HRZ | 22 | ALT>3ULN |
| Abera 2016[55] | Ethiopia | cohort study | May.2014-Oct.2014 | 124 | 34.50 | 46.77 | HRZE | 10 | ALT>3ULN |
| Ben 2016[56] | Tunisia | cohort study | Jan.2013- Sep.2014 | 71 | 36.00 | 69.01 | HRZE | 11 | ALT>2ULN |
| Bouazzi 2016[57] | Morocco | cohort study | Jul.2014-Mar.2015 | 142 | 42.60 | 58.45 | HRZ | 35 | Not clear |
| Carolline 2016[58] | Brazil | cohort study | - | 173 | - | - | HRZE | 53 | ALT>3ULN |
| Isa 2016[59] | Nigeria | cohort study | Jan.2013- Jun.2013 | 110 | 30.00 | 54.55 | HRZES | 20 | ALT>3ULN |
| Kim 2016[60] | Korea | cohort study | Jan.2005-Feb.2014 | 379 | - | - | HRZE | 52 | ALT>3ULN |
| Perwitasari 2016[61] | Indonesia | nested case-control | Jan.2013- Dec.2013 | 55 | - | 52.73 | HRZE | 25 | Not clear |
| Saha 2016[62] | India | cohort study | Jan.2008- Dec.2012 | 253 | - | - | Not clear | 24 | ALT>5ULN |
| Sun 2016[63] | China | cohort study | Mar.2011-Sep.2012 | 938 | - | 58.85 | HRZES | 121 | ALT>3ULN |
| Thomas 2016[64] | UK | cohort study | Jan.1981-Dec.2010 | 2070 | - | 49.81 | HRZE | 63 | ALT>5ULN |
| Vijayalakshi2016[65] | India | cohort study | Sep.2015-Apr.2016 | 100 | - | 63.00 | HRZES | 22 | ALT>2ULN |
| Wu 2016[66] | China | cohort study | Aug.2012-Jan.2016 | 287 | 38.30 | 39.02 | HRZE | 30 | ALT>2ULN |
| Costiniuk 2015[67] | Durban | cohort study | 2005-2013 | 1016 | - | - | HRZ | 53 | ALT>3ULN |
| Erin 2015[68] | USA | nested case-control | Mar.2002- Sep.2009 | 6862 | - | - | H | 77 | ALT>3ULN |
| Fernandes 2015[69] | Brazil | nested case-control | - | 220 | 49.80 | 49.09 | HRZ | 31 | ALT>3ULN |
| Gaude 2015[70] | India | nested case-control | 2009-2013 | 3900 | 47.00 | 66.92 | HRZES | 150 | ALT>5ULN |
| Horita 2015[71] | Japan | case series | Jan.2007-Sep.2012 | 75 | 79.00 | 56.00 | HRE | 18 | ALT>3ULN |
| Horita 2015[71] | Japan | case series | Jan.2007-Sep.2012 | 308 | 60.00 | 70.78 | HRZE | 24 | ALT>3ULN |
| Nooredinvand2015[72] | UK | cohort study | Sep.2008-May.2011 | 429 | 46.40 | 51.28 | HRZE | 28 | ALT>2ULN |
| Sharma 2015[73] | India | cohort study | Jan.2010- Jun.2013 | 67 | 52.00 | 85.07 | HRZE | 24 | ALT>3ULN |
| Tomich 2015[74] | Brazil | cohort study | Aug.2010-Aug.2011 | 148 | 42.00 | 62.84 | HRZ | 33 | ALT>5ULN |
| Wang 2015[75] | China | cohort study | Mar.2010-Feb.2013 | 355 | 57.60 | 65.63 | HRZE | 70 | ALT>3ULN |
| Zazuli 2015[76] | Indonesia | cohort study | - | 106 | - | 67.92 | HRZE | 35 | ALT>2ULN |
| Zhang 2015[77] | China | cohort study | Apr.2009- Oct.2010 | 2091 | 43.10 | 72.26 | HRZE | 205 | Not clear |
| Abbasi 2014[78] | Pakistan | case series | Apr.2012-Mar.2013 | 179 | 36.51 | 55.87 | HRZ | 22 | ALT>3ULN |
| Agha 2014[79] | Egypt | cohort study | Feb.2013- Feb.2014 | 68 | 49.00 | 60.29 | HRZE | 8 | ALT>3ULN |
| Castro 2014[80] | Portugal | cohort study | 2004-2013 | 764 | - | - | HRZES | 53 | ALT>3ULN |
| Chang 2014[81] | USA | cohort study | Jan.2005- Aug.2011 | 1582 | - | 50.13 | H | 13 | ALT>3ULN |
| Hatamkhani 2014[82] | Iran | RCT | Oct.2010-May.2013 | 116 | - | 75.00 | HRZE | 29 | ALT>3ULN |
| Liu 2014[83] | USA | cohort study | Jan.2002- Dec.2009 | 553 | - | 73.42 | HRZE | 72 | ALT>3ULN |
| Mo 2014[84] | China | cohort study | Feb.2012- Feb.2013 | 1374 | - | 73.29 | HRZE | 139 | ALT>3ULN |
| Rana 2014[85] | India | cohort study | - | 300 | - | 61.67 | HRZE | 55 | ALT>2ULN |
| Schultz 2014[86] | Brazil | cohort study | Jan.2000- Dec.2012 | 69 | - | 60.87 | Not clear | 23 | ALT>3ULN |
| Xiang 2014[87] | China | case series | Jan.2010- May.2012 | 2244 | - | 56.11 | HRZES | 89 | ALT>2ULN |
| Zaverucha 2014[88] | Brazil | cohort study | 2001-2008 | 131 | 39.80 | 66.41 | HRZ | 49 | ALT>2ULN |
| Zuberi 2014[89] | Pakistan | cohort study | Dec.2011-Nov.2013 | 325 | - | - | HRZES | 34 | ALT>3ULN |
| Gupta 2013[90] | India | cohort study | Jan.2010-Mar.2012 | 215 | - | 57.21 | HRZE | 50 | ALT>2ULN |
| Hassen 2013[91] | Ethiopia | nested case-control | Jan.2008- Dec.2011 | 288 | - | - | HRZE | 33 | ALT>3ULN |
| Lomtadze 2013[92] | USA | cohort study | Mar.2007-Mar.2010 | 288 | 37.00 | 85.07 | HRZE | 54 | ALT>1.25ULN |
| Santos 2013[93] | Brazil | cohort study | - | 270 | 44.90 | 49.63 | HRZ | 18 | ALT>3ULN |
| Shu 2013 [94] | China | cohort study | Jan.2005- Dec.2009 | 926 | - | 67.82 | HRZ | 111 | ALT>3ULN |
| Babalik 2012[95] | Turkey | cohort study | Jan.2004- Dec.2007 | 1443 | 38.37 | 64.52 | HRZES | 106 | ALT>3ULN |
| Keshavjee 2012[96] | USA | cohort study | Sep.2000-Nov.2004 | 568 | 34.00 | 82.22 | Not clear | 91 | ALT>3ULN |
| Mansukhani 2012[97] | India | cohort study | Apr.2007-Feb.2008 | 46 | 5.88 | - | HRZE | 7 | ALT>3ULN |
| Monteiro 2012[98] | Brazil | cohort study | 2006-2011 | 177 | 37.00 | 66.10 | Not clear | 59 | ALT>2ULN |
| Nanashima 2012[99] | Japan | nested case-control | 2003-2005 | 100 | 64.00 | 56.00 | RZ | 18 | ALT>2ULN |
| Singanayagam 2012[100] | UK | cohort study | Jan.2007-Sep.2009 | 288 | - | 53.82 | Not clear | 21 | ALT>3ULN |
| Thongraung 2012[101] | Thailand | case series | Oct.2006-Sep.2009 | 625 | 51.70 | - | HRZES | 45 | ALT>3ULN |
| Bose 2011[102] | India | cohort study | - | 218 | 36.00 | 46.79 | HRZE | 41 | ALT>2ULN |
| Leiro 2011[103] | Spain | nested case-control | Jan.1998-Dec.2008 | 1200 | - | - | HRZ | 50 | ALT>3ULN |
| Lorent 2011[104] | Canada | cohort study | May.2008-Jan.2010 | 253 | 35.00 | 58.50 | HRZE | 24 | ALT>3ULN |
| Mankhatitham 2011[105] | Thailand | RCT | Dec.2006-Oct.2007 | 134 | 36.80 | 67.16 | Not clear | 9 | ALT>2ULN |
| Shang 2011[106] | China | cohort study | Oct.2007-Jun.2008 | 4304 | 42.00 | 71.61 | HRZES | 106 | ALT>3ULN |
| Teixeira 2011[107] | Brazil | nested case-control | 1998-2008 | 167 | - | - | H | 26 | ALT>3ULN |
| Baghaei 2010[108] | Iran | cohort study | Jan.2006- Jan.2008 | 761 | 52.44 | 47.96 | HRZE | 99 | Not clear |
| Coca 2010[109] | Brazil | case series | 2005-2007 | 162 | - | 72.22 | HRZ | 17 | ALT>3ULN |
| Khoharo 2010[110] | Pakistan | cohort study | Jul.2007-Aug.2008 | 350 | 34.62 | 55.71 | HRZES | 91 | ALT>5ULN |
| Lee 2010[111] | China | cohort study | 2007-2008 | 140 | - | 62.29 | HRZE | 45 | ALT>2ULN |
| Moses 2010[112] | UK | cohort study | Jun.2007-Dec.2007 | 156 | 35.00 | 54.49 | HRZE | 3 | ALT>2.5ULN |
| Nader 2010[113] | Brazil | cohort study | 1998-2006 | 534 | 39.08 | 75.09 | HRZ | 47 | ALT>3ULN |
| Naz 2010[114] | Pakistan | cohort study | Aug,2007-Jun.2008 | 50 | - | 28.00 | HRZES | 5 | ALT>5ULN |
| Park 2010[115] | Korea | cohort study | Jan.2000-Jul.2008 | 107 | 51.00 | 75.70 | HRZ | 18 | ALT>3ULN |
| Sharma 2010[116] | India | RCT | 2004-2009 | 175 | - | 48.57 | HRZ | 19 | ALT>5ULN |
| Tedla 2010[117] | Botswana | cohort study | 2004-2006 | 1995 | 32.00 | 26.17 | H | 19 | Not clear |
| Tostmann 2010[118] | Netherlands | cohort study | Apr.2007-Jun.2008 | 112 | 32.00 | 78.57 | HRZE | 10 | ALT>3ULN |
| Fountain 2009[119] | USA | cohort study | Jun.2001-May.2007 | 205 | - | 67.80 | R | 4 | ALT>3ULN |
| Walker 2009[120] | UK | nested case-control | Jun.2006-Jun.2007 | 94 | 43.00 | - | Not clear | 14 | ALT>3ULN |
| Kim 2009[121] | Korea | case series | Jul.2003-Aug.2007 | 837 | - | - | HRZE | 67 | ALT>2ULN |
| Chang 2008[122] | China | nested case-control | Jan.2001-Jun.2001 | 3007 | - | 64.95 | HRZE | 150 | ALT>3ULN |
| Makhlouf 2008[123] | Egypt | cohort study | Oct.2004-Jan.2005 | 100 | 33.60 | 44.00 | HRZES | 15 | ALT>2ULN |
| Marzuki 2008[124] | Malaysia | cohort study | Jan.2003-Jun.2005 | 473 | - | - | HRZ | 46 | ALT>3ULN |
| Possuelo 2008[125] | Brazil | cohort study | Aug.2005-Jun.2007 | 254 | 36.50 | 66.93 | HRZ | 14 | ALT>3ULN |
| Yimer 2008[126] | Ethiopia | cohort study | Aug.2004-Mar.2005 | 197 | 26.00 | 53.30 | HRZES | 8 | ALT>5ULN |
| Cho 2007[127] | Korea | cohort study | Jun.2004-Dec.2005 | 132 | - | 56.82 | HRZE | 18 | ALT>2ULN |
| Kwon 2007[128] | Korea | case series | Sep.1994-Jun.2005 | 54 | 56.40 | 61.11 | HRZE | 7 | ALT>3ULN |
| Mahmood 2007[129] | Pakistan | cohort study | Jul.2004-Jul.2005 | 339 | 36.00 | 53.98 | HRZES | 67 | ALT>5ULN |
| Pukenyte 2007[130] | French | cohort study | Jan.1992-Dec.2004 | 144 | - | 76.39 | HRZE | 15 | ALT>5ULN |
| Cook 2006[131] | USA | cohort study | Jun.2000-Jan.2006 | 310 | 39.30 | 52.26 | RZ | 57 | ALT>1.25ULN |
| Cook 2006[131] | USA | cohort study | Jun.2000-Jan.2006 | 149 | 37.60 | 54.36 | H | 17 | ALT>1.25ULN |
| Gülbay 2006[132] | Japan | cohort study | 1984-2001 | 1149 | 36.10 | 76.24 | HRZES | 28 | ALT>3ULN |
| Senaratne 2006[133] | Sri Lanka | cohort study | Apr.2001- Apr.2002 | 783 | 45.20 | 72.16 | HRZE | 74 | ALT>3ULN |
| Vuilleumier 2006[134] | Switzerland | cohort study | Oct.2001-Mar.2003 | 89 | 31.00 | 55.06 | H | 8 | ALT>4ULN |
| Fountain 2005[135] | USA | cohort study | Jul.1996-Dec.2003 | 3377 | - | 61.45 | H | 19 | AST>5ULN |
| Meier 2005[136] | Switzerland | cohort study | Jan.1996-Dec.2000 | 4209 | 61.00 | 58.02 | Not clear | 88 | ALT>2ULN |
| Shakya 2005[137] | Germany | cohort study | Nov.2001-Oct,2002 | 50 | 30.50 | 56.00 | HRZE | 4 | ALT>5ULN |
| Sharifzadeh 2005[138] | Iran | cohort study | 1999-2002 | 112 | - | 53.57 | HRZE | 31 | ALT>3ULN |
| Kandula 2004[139] | USA | cohort study | Aug.2001-Oct.2001 | 34 | - | - | RZ | 5 | ALT>5ULN |
| Menzies 2004[140] | Canada | RCT | Jan.2002-Oct.2002 | 52 | - | - | H | 3 | Not clear |
| Pedral 2004[141] | Brazil | cohort study | 1999-2002 | 49 | 34.70 | 69.39 | HRZ | 6 | Not clear |
| Huang 2003[142] | China | cohort study | May.1998-Aug.2001 | 318 | - | 15.41 | HRZE | 49 | ALT>2ULN |
| LoBue 2003[143] | USA | cohort study | Jul.1999-Nov.2002 | 3788 | - | - | H | 10 | Not clear |
| Stout 2003[144] | USA | cohort study | Dec.1999-Apr.2002 | 114 | 43.00 | 80.70 | RZ | 6 | ALT>5ULN |
| Yee 2003[145] | Canada | cohort study | 1990-2000 | 408 | 40.30 | - | HRZ | 12 | ALT>3ULN |
| Jasmer 2002[146] | USA | RCT | Feb.1999-Oct.2000 | 207 | - | - | RZ | 54 | ALT>1.25ULN |
| Jasmer 2002[146] | USA | RCT | Feb.1999-Oct.2000 | 204 | - | - | H | 32 | ALT>1.25ULN |
| Lee 2002[147] | USA | cohort study | Apr.1999-Mar.2001 | 148 | 37.00 | 56.76 | RZ | 14 | ALT>1ULN |
| Ohkawa 2002[148] | Japan | cohort study | Jan.1995-Nov.1999 | 99 | - | - | HRZES | 8 | ALT>5ULN |
| Teleman 2002[149] | Singapore | cohort study | Jan.1998-Dec.1998 | 1036 | - | 72.39 | HRZ(+E/S) | 55 | ALT>3ULN |
| Ohno 2000[150] | Japan | cohort study | 1996-1998 | 77 | 51.00 | 59.74 | HR(+E/S) | 14 | ALT>2ULN |
| Ungo 1998[151] | USA | cohort study | Dec.1994-May.1996 | 128 | 44.00 | 75.00 | HRZ | 22 | ALT>3ULN |
| Hwang 1997[152] | China | cohort study | Aug.1991-Jul.1994 | 240 | 40.00 | 64.17 | HRZE | 20 | Not clear |
| Martin 1996[153] | Denmark | cohort study | Jan.1983-Jan.1994 | 752 | 45.00 | 64.50 | HRZE | 61 | AST>2ULN |
| Ozick 1995[154] | USA | cohort study | May.1990-Nov.1990 | 70 | 38.90 | 82.86 | HR | 8 | ALT>5ULN |
| Barazzone 1993[155] | Switzerland | cohort study | Oct.1989-Oct.1991 | 43 | 6.00 | 46.51 | HRZE | 2 | ALT>3ULN |
| Aziz1990[156] | Pakistan | cohort study | - | 47 | 29.00 | 74.47 | HRZES | 7 | Not clear |

TB, Tuberculosis; ATLI, anti-tuberculosis drug-induced liver injury; ALT, alanine transaminase; AST, aspartate aminotransferase; USA, United States of America; UK, the United Kingdom; RCT, Randomized Controlled Trial; H, Isoniazid; R, Rifampin; E, Ethambutol; Z, Pyrazinamide; S, Streptomycin.

Supplemental Table 3. Summary of risk of bias assessment

| Study | 1.Study participation | 2.Study attrition | 3.Outcome measurement | 4. Causality assessment | Overall quality assessment |
| --- | --- | --- | --- | --- | --- |
| Chbili 2022[1] | moderate | moderate | low | high | moderate |
| Chong 2022[2] | low | low | low | moderate | low |
| Maddahi 2022[3] | low | low | moderate | high | moderate |
| Nie 2022[4] | low | low | low | high | low |
| Ruslami2022[5] | low | low | low | high | low |
| Ai 2021[6] | moderate | moderate | moderate | high | moderate |
| Devrim 2021[7] | low | moderate | low | high | moderate |
| Herrera 2021[8] | moderate | moderate | low | high | moderate |
| Jiang 2021[9] | moderate | low | low | low | low |
| Khiewkhern 2021[10] | moderate | moderate | moderate | high | moderate |
| Liu 2021[11] | moderate | moderate | low | high | moderate |
| Molla 2021[12] | moderate | moderate | low | high | moderate |
| Su 2021[13] | moderate | moderate | low | high | moderate |
| Wang 2021[14] | low | low | moderate | high | moderate |
| Xiong 2021[15] | moderate | low | low | high | moderate |
| Zhong 2021[16] | low | moderate | moderate | high | high |
| Ali 2020[17] | moderate | high | moderate | high | high |
| Gezahegn 2020[18] | low | low | low | high | low |
| Ho 2020[19] | low | low | low | moderate | low |
| Kesenogile 2020[20] | low | low | low | low | low |
| Mo 2020[21] | moderate | low | low | moderate | low |
| Patterson 2020[22] | low | moderate | moderate | high | moderate |
| Ronald 2020[23] | moderate | moderate | low | high | moderate |
| Subbalaxmi 2020[24] | moderate | low | low | high | moderate |
| Yang 2020[25] | moderate | moderate | low | low | moderate |
| Zeleke 2020[26] | high | low | moderate | high | high |
| Zhao 2020[27] | low | high | low | high | moderate |
| Bai 2019[28] | low | low | moderate | high | moderate |
| Chang 2019[29] | moderate | low | low | high | moderate |
| Chen 2019[30] | moderate | high | low | low | moderate |
| Dawra 2019[31] | high | moderate | low | high | high |
| Gafar 2019[32] | moderate | moderate | low | high | moderate |
| Indumathi 2019[33] | high | low | high | high | high |
| Lang 2019[34] | high | low | moderate | high | high |
| Laoveeravat2019[35] | high | low | moderate | high | high |
| Naidoo 2019[36] | low | low | low | high | low |
| Santos 2019[37] | low | moderate | low | high | moderate |
| Song 2019[38] | low | low | low | high | low |
| Zhang 2019[39] | moderate | moderate | low | high | moderate |
| Cheah 2018[40] | moderate | low | low | high | moderate |
| Chua 2018[41] | high | low | moderate | high | high |
| Tersigni 2018[42] | low | low | low | high | low |
| Tweed 2018[43] | high | low | low | moderate | low |
| Wang 2018[44] | moderate | low | low | moderate | low |
| Abbara 2017[45] | moderate | low | low | moderate | low |
| Chamorro 2017[46] | low | low | low | high | low |
| Cusack 2017[47] | high | low | high | high | high |
| Hasanain 2017[48] | moderate | low | low | high | moderate |
| Kumar 2017[49] | moderate | low | low | high | moderate |
| Latief 2017[50] | high | moderate | moderate | high | moderate |
| Nataprawira 2017[51] | high | low | high | high | high |
| Petros 2017[52] | moderate | low | low | moderate | low |
| Sekaggya 2017[53] | moderate | low | low | high | moderate |
| Zhu 2017[54] | low | low | low | high | low |
| Abera 2016[55] | low | low | low | high | low |
| Ben 2016[56] | low | low | low | moderate | low |
| Bouazzi 2016[57] | moderate | low | low | high | moderate |
| Carolline 2016[58] | high | low | low | high | moderate |
| Isa 2016[59] | low | low | low | high | low |
| Kim 2016[60] | moderate | low | low | high | moderate |
| Perwitasari 2016[61] | moderate | low | high | high | high |
| Saha 2016[62] | moderate | low | low | high | moderate |
| Sun 2016[63] | low | moderate | low | moderate | low |
| Thomas 2016[64] | moderate | low | low | high | moderate |
| Vijayalakshi2016[65] | moderate | low | high | high | high |
| Wu 2016[66] | low | moderate | low | low | low |
| Costiniuk 2015[67] | high | low | moderate | high | high |
| Erin 2015[68] | moderate | low | low | high | moderate |
| Fernandes 2015[69] | high | low | moderate | high | high |
| Gaude 2015[70] | moderate | low | low | high | moderate |
| Horita 2015[71] | moderate | low | low | high | moderate |
| Nooredinvand2015[72] | moderate | low | low | high | moderate |
| Sharma 2015[73] | high | low | moderate | high | high |
| Tomich 2015[74] | moderate | low | low | high | moderate |
| Wang 2015[75] | low | low | low | high | low |
| Zazuli 2015[76] | high | low | low | high | moderate |
| Zhang 2015[77] | high | low | high | moderate | moderate |
| Abbasi 2014[78] | high | low | moderate | high | high |
| Agha 2014[79] | low | low | low | high | low |
| Castro 2014[80] | high | low | moderate | high | high |
| Chang 2014[81] | moderate | low | low | high | moderate |
| Hatamkhani 2014[82] | moderate | low | low | moderate | low |
| Liu 2014[83] | moderate | low | low | high | moderate |
| Mo 2014[84] | moderate | low | low | high | moderate |
| Rana 2014[85] | high | low | low | high | moderate |
| Schultz 2014[86] | high | low | moderate | high | high |
| Xiang 2014[87] | low | low | low | high | low |
| Zaverucha 2014[88] | moderate | low | low | high | moderate |
| Zuberi 2014[89] | high | low | moderate | high | high |
| Gupta 2013[90] | low | low | low | high | low |
| Hassen 2013[91] | high | moderate | moderate | moderate | moderate |
| Lomtadze 2013[92] | moderate | low | low | high | moderate |
| Santos 2013[93] | moderate | low | low | high | moderate |
| Shu 2013 [94] | moderate | low | low | high | moderate |
| Babalik 2012[95] | high | low | moderate | high | high |
| Keshavjee 2012[96] | low | low | low | high | low |
| Mansukhani 2012[97] | high | low | moderate | high | high |
| Monteiro 2012[98] | moderate | low | low | high | moderate |
| Nanashima 2012[99] | low | low | low | high | low |
| Singanayagam 2012[100] | low | low | low | high | low |
| Thongraung 2012[101] | moderate | moderate | low | high | moderate |
| Bose 2011[102] | moderate | low | low | high | moderate |
| Leiro 2011[103] | moderate | low | low | high | moderate |
| Lorent 2011[104] | moderate | moderate | moderate | high | moderate |
| Mankhatitham 2011[105] | moderate | low | low | high | moderate |
| Shang 2011[106] | low | moderate | low | low | low |
| Teixeira 2011[107] | moderate | low | low | high | moderate |
| Baghaei 2010[108] | moderate | low | high | high | high |
| Coca 2010[109] | moderate | high | low | high | high |
| Khoharo 2010[110] | high | low | moderate | high | high |
| Lee 2010[111] | low | low | low | high | low |
| Moses 2010[112] | moderate | low | low | high | moderate |
| Nader 2010[113] | moderate | low | moderate | high | moderate |
| Naz 2010[114] | moderate | low | moderate | high | moderate |
| Park 2010[115] | high | low | moderate | high | high |
| Sharma 2010[116] | high | high | moderate | high | high |
| Tedla 2010[117] | moderate | low | high | high | high |
| Tostmann 2010[118] | moderate | moderate | low | high | moderate |
| Fountain 2009[119] | moderate | low | low | high | moderate |
| Walker 2009[120] | moderate | moderate | low | high | moderate |
| Kim 2009[121] | moderate | moderate | low | high | moderate |
| Chang 2008[122] | moderate | low | low | high | moderate |
| Makhlouf 2008[123] | low | low | low | high | low |
| Marzuki 2008[124] | high | low | moderate | high | high |
| Possuelo 2008[125] | moderate | low | low | high | moderate |
| Yimer 2008[126] | moderate | low | low | high | moderate |
| Cho 2007[127] | moderate | low | low | high | moderate |
| Kwon 2007[128] | moderate | low | low | high | moderate |
| Mahmood 2007[129] | high | low | moderate | high | high |
| Pukenyte 2007[130] | moderate | low | moderate | high | moderate |
| Cook 2006[131] | moderate | low | low | high | moderate |
| Gülbay 2006[132] | low | low | low | high | low |
| Senaratne 2006[133] | low | moderate | low | high | moderate |
| Vuilleumier 2006[134] | low | low | low | low | low |
| Fountain 2005[135] | moderate | low | low | high | moderate |
| Meier 2005[136] | low | low | low | low | low |
| Shakya 2005[137] | high | low | moderate | high | high |
| Sharifzadeh 2005[138] | high | low | moderate | moderate | moderate |
| Kandula 2004[139] | moderate | low | low | high | moderate |
| Menzies 2004[140] | high | moderate | high | high | high |
| Pedral 2004[141] | high | low | high | high | high |
| Huang 2003[142] | low | low | low | low | low |
| LoBue 2003[143] | high | low | high | high | high |
| Stout 2003[144] | moderate | low | moderate | high | moderate |
| Yee 2003[145] | moderate | low | low | high | moderate |
| Jasmer 2002[146] | moderate | high | low | high | high |
| Lee 2002[147] | low | low | low | high | low |
| Ohkawa 2002[148] | high | moderate | moderate | high | high |
| Teleman 2002[149] | moderate | low | moderate | high | moderate |
| Ohno 2000[150] | high | low | moderate | high | high |
| Ungo 1998[151] | moderate | moderate | low | high | moderate |
| Hwang 1997[152] | high | low | moderate | moderate | moderate |
| Martin 1996[153] | moderate | moderate | low | high | moderate |
| Ozick 1995[154] | low | high | low | high | moderate |
| Barazzone 1993[155] | moderate | moderate | low | high | moderate |
| Aziz1990[156] | high | high | high | high | high |

Supplemental Table 4. The pooled incidence of ATLI in different countries

| Country | ATLI incidence (%) | Country | ATLI incidence (%) |
| --- | --- | --- | --- |
| Argentina | 27.83 | Malaysia | 8.79 |
| Botswana | 5.30 | Mexico | 7.69 |
| Brazil | 17.44 | Morocco | 24.65 |
| Canada | 2.27 | Netherlands | 11.85 |
| China | 13.04 | Nigeria | 18.18 |
| Denmark | 8.11 | Pakistan | 15.61 |
| Durban | 5.22 | Portugal | 6.94 |
| Egypt | 11.03 | Singapore | 4.34 |
| Ethiopia | 10.11 | Spain | 4.17 |
| French | 10.42 | Sri Lanka | 9.45 |
| Germany | 9.25 | Sweden | 24.24 |
| India | 12.86 | Switzerland | 4.50 |
| Indonesia | 24.51 | Thailand | 6.77 |
| Iran | 21.72 | Tunisia | 19.53 |
| Ireland | 5.45 | Turkey | 4.44 |
| Italy | 1.13 | Uganda | 35.07 |
| Japan | 11.57 | United Kingdom | 5.71 |
| Korea | 12.22 | United States of America | 10.06 |

ATLI, anti-tuberculosis drug-induced liver injury.

**References**

1. Chbili, C., et al., Glutathione-S-transferase genetic polymorphism and risk of hepatotoxicity to antitubercular drugs in a North-African population: A case-control study. Gene, 2022. 809: p. 146019. DOI: 10.1016/j.gene.2021.146019.
2. Chong, Y., et al., Interaction between the HIF-1α gene rs1957757 polymorphism and CpG island methylation in the promoter region is associated with the risk of anti-tuberculosis drug-induced liver injury in humans: A case-control study. J Clin Pharm Ther, 2022. DOI: 10.1111/jcpt.13625.
3. Maddahi, S.Z., et al., The efficacy of Jujube syrup on the prevention of drug-induced hepatotoxicity in pulmonary tuberculosis patients: A pilot randomized double-blind placebo-controlled clinical trial. Pharmacol Res Perspect, 2022. 10(1): p. e00902. DOI: 10.1002/prp2.902.
4. Nie, Q., et al., High-dose gatifloxacin-based shorter treatment regimens for MDR/RR-TB. Int J Infect Dis, 2022. 115: p. 142-148. DOI: 10.1016/j.ijid.2021.11.037.
5. Ruslami, R., et al., Pharmacokinetics and safety/tolerability of isoniazid, rifampicin and pyrazinamide in children and adolescents treated for tuberculous meningitis. Arch Dis Child, 2022. 107(1): p. 70-77. DOI: 10.1136/archdischild-2020-321426.
6. Ai, X., et al., Relationship between xanthine oxidase gene polymorphisms and anti-tuberculosis drug-induced liver injury in a Chinese population. Infect Genet Evol, 2021. 93: p. 104991.DOI: 10.1016/j.meegid.2021.104991.
7. Devrim, İ., et al., Isoniazid-induced hepatotoxicity in children with latent tuberculosis infection. Minerva Pediatr (Torino), 2021. 73(2): p. 184-187.DOI: 10.23736/s2724-5276.17.04338-9.
8. Herrera-Rodulfo, A., et al., NAT2 polymorphisms associated with the development of hepatotoxicity after first-line tuberculosis treatment in Mexican patients: From genotype to molecular structure characterization. Clin Chim Acta, 2021. 519: p. 153-162.DOI: 10.1016/j.cca.2021.04.017.
9. Jiang, F., et al., Incidence and risk factors of anti-tuberculosis drug-induced liver injury (DILI): Large cohort study involving 4652 Chinese adult tuberculosis patients. Liver Int, 2021. 41(7): p. 1565-1575.DOI: 10.1111/liv.14896.
10. Khiewkhern, S., et al., The Information from Medical Data Based: Prevalence and Expected Median Survival Time of Drug-Induced Hepatotoxicity Among Thai Patients with TB. Stud Health Technol Inform, 2021. 281: p. 784-788.DOI: 10.3233/shti210282.
11. Liu, Y.H., et al., Impact of Non-Alcoholic Simple Fatty Liver Disease on Antituberculosis Drug-Induced Liver Injury. Infect Drug Resist, 2021. 14: p. 3667-3671.DOI: 10.2147/idr.S326386.
12. Molla, Y., M. Wubetu, and B. Dessie, Anti-Tuberculosis Drug Induced Hepatotoxicity and Associated Factors among Tuberculosis Patients at Selected Hospitals, Ethiopia. Hepat Med, 2021. 13: p. 1-8.DOI: 10.2147/hmer.S290542.
13. Su, Q., et al., Study on the associations between liver damage and antituberculosis drug rifampicin and relative metabolic enzyme gene polymorphisms. Bioengineered, 2021. 12(2): p. 11700-11708.DOI: 10.1080/21655979.2021.2003930.
14. Wang, J., et al., Dietary Intake of Vegetables and Cooking Oil Was Associated With Drug-Induced Liver Injury During Tuberculosis Treatment: A Preliminary Cohort Study. Front Nutr, 2021. 8: p. 652311.DOI: 10.3389/fnut.2021.652311.
15. Xiong, K., et al., Vitamins A and D fail to protect against tuberculosis-drug-induced liver injury: A post hoc analysis of a previous randomized controlled trial. Nutrition, 2021. 86: p. 111155.DOI: 10.1016/j.nut.2021.111155.
16. Zhong, T., et al., Predicting Antituberculosis Drug-Induced Liver Injury Using an Interpretable Machine Learning Method: Model Development and Validation Study. JMIR Med Inform, 2021. 9(7): p. e29226.DOI: 10.2196/29226.
17. Ali N, Gupta N, Saravu K. Malnutrition as an important risk factor for drug-induced liver injury in patients on anti-tubercular therapy: an experience from a tertiary care center in South India. Drug discoveries & therapeutics 2020; 14(3):135-138. DOI: 10.5582/ddt.2020.03029.
18. Gezahegn, L.K., et al., Magnitude, outcome, and associated factors of anti-tuberculosis drug-induced hepatitis among tuberculosis patients in a tertiary hospital in North Ethiopia: A cross-sectional study. PLoS One, 2020. 15(11): p. e0241346.DOI: 10.1371/journal.pone.0241346.
19. Ho, C.M., et al., Circulatory Inflammatory Mediators in the Prediction of Anti-Tuberculous Drug-Induced Liver Injury Using RUCAM for Causality Assessment. Biomedicines, 2021. 9(8).DOI: 10.3390/biomedicines9080891.
20. Kesenogile, B., B. Godman, and G.M. Rwegerera, Alanine transaminase and hemoglobin appear to predict the occurrence of antituberculosis medication hepatotoxicity; findings and implications in Botswana. Expert Rev Anti Infect Ther, 2021. 19(3): p. 379-391.DOI: 10.1080/14787210.2020.1822735.
21. Mo X, Xu X, Ren Z, Guan J, Peng J. Patients with tuberculous meningitis and hepatitis B co-infection have increased risk for antituberculosis drug-induced liver injury and poor outcomes. Infectious diseases (London, England) 2020;0(0):1-7. DOI: 10.1080/23744235.2020.1788223.
22. Patterson, B., et al., Predicting drug-induced liver injury from anti-tuberculous medications by early monitoring of liver tests. J Infect, 2021. 82(2): p. 240-244.DOI: 10.1016/j.jinf.2020.09.038.
23. Ronald, L.A., et al., Treatment with isoniazid or rifampin for latent tuberculosis infection: population-based study of hepatotoxicity, completion and costs. Eur Respir J, 2020. 55(3).DOI: 10.1183/13993003.02048-2019.
24. Soanker, R. and A.V. Lakshmi, Evaluation of Risk Factors for Development of Anti-Tubercular Therapy Induced Hepatotoxicity: A Prospective Study. Curr Drug Saf, 2020. 15(3): p. 198-204.DOI: 10.2174/1574886315666200626164554.
25. Yang M, Pan H, Chen H, Liu W, Lu L, He X, et al. Association between NR1I2 polymorphisms and susceptibility to anti-tuberculosis drug-induced hepatotoxicity in an Eastern Chinese Han population: A case-control study. Infection, genetics and evolution, journal of molecular epidemiology and evolutionary genetics in infectious diseases 2020; 83.DOI: 10.1016/j.meegid.2020.104349.
26. Zeleke A, Misiker B, Yesuf TA. Drug-induced hepatotoxicity among TB/HIV co-infected patients in a referral hospital, Ethiopia. BMC research notes 2020; 13(1):2.DOI: 10.1186/s13104-019-4872-1.
27. Zhao Z, Peng W, Wu L, Ying B. Correlation between lncRNA AC079767.4 variants and liver injury from antituberculosis treatment in West China. Journal of infection and chemotherapy: official journal of the Japan Society of Chemotherapy 2020; 26(1):63-68.DOI: 10.1016/j.jiac.2019.07.003.
28. Bai, H., et al., Association of ABCC Gene Polymorphism With Susceptibility to Antituberculosis Drug-Induced Hepatotoxicity in Western Han Patients With Tuberculosis. J Clin Pharmacol, 2020. 60(3): p. 361-368.DOI: 10.1002/jcph.1533.
29. Chang TE, Huang YS, Su WJ, Perng CL, Huang YH, Hou MC. The role of regular liver function monitoring in antituberculosis drug-induced liver injury. Journal of the Chinese Medical Association 2019; 82(7):535-540. DOI: 10.1097/JCMA.0000000000000119.
30. Chen S, Pan H, Chen Y, Lu L, He X, Chen H, et al. Association between genetic polymorphisms of NRF2, KEAP1, MAFF, MAFK and anti-tuberculosis drug-induced liver injury: a nested case-control study. Scientific reports 2019; 9(1):14311.DOI: 10.1038/s41598-019-50706-y.
31. Dawra S, Mandavdhare HS, Singh H, Prasad KK, Dutta U, Sharma V. Extra-abdominal involvement is associated with antitubercular therapy-related hepatitis in patients treated for abdominal tuberculosis. Clinical and experimental hepatology 2019; 5(1):60-64.DOI: 10.5114/ceh.2019.83158.
32. Gafar F, Arifin H, Jurnalis YD, Yani FF, Fitria N, Alffenaar JC, et al. Antituberculosis Drug-induced Liver Injury in Children: Incidence and Risk Factors During the Two-month Intensive Phase of Therapy. The Pediatric infectious disease journal 2019; 38(1):50-53.DOI: 10.1097/inf.0000000000002192.
33. Indumathi CK, Sethuraman A, Jain S, Krishnamurthy S. Revised Antituberculosis Drug Doses and Hepatotoxicity in HIV Negative Children. Indian journal of pediatrics 2019; 86(3):229-232.DOI: 10.1007/s12098-018-2812-z.
34. Lang SM, Ortmann J, Rostig S, Schiffl H. Ursodeoxycholic acid attenuates hepatotoxicity of multidrug treatment of mycobacterial infections: A prospective pilot study. International journal of mycobacteriology 2019; 8(1):89-92. DOI: 10.4103/ijmy.ijmy_159_18.
35. Laoveeravat P, Wongjarupong N, Phathong C, Hurst C, Treeprasertsuk S, Rerknimitr R, et al. Characteristics and risk factors for antituberculosis drug-induced liver injury in a cohort of patients with cirHRosis in a tertiary referral university teaching hospital in Thailand. Asian Biomedicine 2019; 12(2):65-74.DOI: 10.1515/abm-2019-0003.
36. Naidoo, K., et al., High Rates of Drug-induced Liver Injury in People Living With HIV Coinfected With Tuberculosis (TB) Irrespective of Antiretroviral Therapy Timing During Antituberculosis Treatment: Results From the Starting Antiretroviral Therapy at Three Points in TB Trial. Clin Infect Dis, 2020. 70(12): p. 2675-2682.DOI: 10.1093/cid/ciz732.
37. Santos EA, Gonçalves JCS, Fleury MK, Kritski AL, Oliveira MM, Velasque LS, et al. Relationship of anti-tuberculosis drug-induced liver injury and genetic polymorphisms in CYP2E1 and GST. Brazilian Journal of Infectious Diseases 2019; 23(6):381-387.DOI: 10.1016/j.bjid.2019.09.003.
38. Song JH, Yoon SY, Park TY, Heo EY, Kim DK, Chung HS, et al. The clinical impact of drug-induced hepatotoxicity on anti-tuberculosis therapy: a case control study. Respiratory research 2019; 20(1):283.DOI: 10.1186/s12931-019-1256-y.
39. Zhang J, Zhao Z, Bai H, Jiao L, Wu Q, Wu T, et al. The Variant at TGFBRAP1 but Not TGFBR2 Is Associated with Antituberculosis Drug-Induced Liver Injury. Evidence-based complementary and alternative medicine 2019.DOI: 10.1155/2019/1685128.
40. Cheah MF, Zainal H, Hyder Ali IA. Evaluation of adverse reactions induced by anti-tuberculosis drugs in hospital Pulau Pinang. Malaysian Journal of Medical Sciences 2018; 25(5):103-114. DOI: 10.21315/mjms2018.25.5.10.
41. Chua APG, Lim LKY, Gan SH, Chee CBE, Wang YT. The role of chronic viral hepatitis on tuberculosis treatment interruption. The international journal of tuberculosis and lung disease: the official journal of the International Union against Tuberculosis and Lung Disease 2018; 22(12):1486-1494. DOI: 10.5588/ijtld.18.0195.
42. Tersigni C, Venturini E, Cordola C, Piccini P, Bianchi L, Montagnani C, et al. Latent tuberculosis in childhood: tolerability of two different therapeutic approaches. Expert review of anti-infective therapy 2018; 16(4):359-365. DOI: 10.1080/14787210.2018.1441025.
43. Tweed CD, Wills GH, Crook AM, Dawson R, Diacon AH, Louw CE, et al. Liver toxicity associated with tuberculosis chemotherapy in the REMoxTB study. BMC medicine 2018; 16(1):46. DOI: 10.1186/s12916-018-1033-7.
44. Wang S, Shangguan Y, Ding C, Li P, Ji Z, Shao J, et al. Risk factors for acute liver failure among inpatients with anti-tuberculosis drug-induced liver injury. The Journal of international medical research 2018; 48(1):1-10. DOI: 10.1177/0300060518811512.
45. Abbara A, Chitty S, Roe JK, Ghani R, Collin SM, Ritchie A, et al. Drug-induced liver injury from antituberculous treatment: a retrospective study from a large TB centre in the UK. BMC infectious diseases 2017; 17(1):231. DOI: 10.1186/s12879-017-2330-z.
46. Chamorro JG, Castagnino JP, Aidar O, Musella RM, Frías A, Visca M, et al. Effect of gene-gene and gene-environment interactions associated with antituberculosis drug-induced hepatotoxicity. Pharmacogenetics and Genomics 2017; 27(10):363-371. DOI: 10.1097/FPC.0000000000000300.
47. Cusack RP, Chawke L, O'Brien DJ, O'Connor B, O'Connor TM. Predictors of hepatotoxicity among patients treated with antituberculous medication. QJM : monthly journal of the Association of Physicians 2017; 110(4):219-225. DOI: 10.1093/qjmed/hcw160.
48. Hasanain AFA, Zayed AAH, Mahdy RE, Nafee AMA. Cholecalciferol for prophylaxis against antituberculosis therapy-induced liver disorders among naïve patients with pulmonary tuberculosis: A randomized, comparative study. Int J Mycobacteriol 2017; 6(2):149-155. DOI: 10.4103/ijmy.ijmy_19_17.
49. Kumar M, Kalita J, Tripathi A, Misra UK. Is drug-induced hepatitis related to the severity of tuberculous meningitis? Transactions of the Royal Society of Tropical Medicine and Hygiene 2017; 111(11):520-526. DOI: 10.1093/trstmh/try006.
50. Latief M, Dar WR, Sofi N, Dar IA, Kasana B, Hussain M, et al. Novel risk factors and early detection of anti-tubercular treatment induced liver injury-Looking beyond American Thoracic Society Guidelines. The Indian journal of tuberculosis 2017; 64(1):26-32. DOI: 10.1016/j.ijtb.2016.11.002.
51. Nataprawira HM, Hannah RA, Kartika HH. Hospitalized pediatric antituberculosis drug induced hepatotoxicity: Experience of an Indonesian referral hospital. Asian Pacific Journal of Tropical Disease 2017; 7(5):276-279. DOI: 10.12980/apjtd.7.2017D6-402.
52. Petros Z, Kishikawa J, Makonnen E, Yimer G, Habtewold A, Aklillu E. HLA-B*57 Allele Is Associated with Concomitant Anti-tuberculosis and Antiretroviral Drugs Induced Liver Toxicity in Ethiopians. Frontiers in pharmacology 2017; 8:90. DOI: 10.3389/fphar.2017.00090.
53. Sekaggya-Wiltshire C, von Braun A, Scherrer AU, Manabe YC, Buzibye A, Muller D, et al. Anti-TB drug concentrations and drug-associated toxicities among TB/HIV-coinfected patients. The Journal of antimicrobial chemotherapy 2017; 72(4):1172-1177. DOI: 10.1093/jac/dkw534.
54. Zhu CH, Zhao MZ, Chen G, Qi JY, Song JX, Ning Q, et al. Baseline HBV load increases the risk of anti-tuberculous drug-induced hepatitis flares in patients with tuberculosis. Journal of Huazhong University of Science and Technology Medical scien 2017; 37(1):105-109. DOI: 10.1007/s11596-017-1702-3.
55. Abera W, Cheneke W, Abebe G. Incidence of antituberculosis-drug-induced hepatotoxicity and associated risk factors among tuberculosis patients in Dawro Zone, South Ethiopia: A cohort study. International Journal of Mycobacteriology 2016; 5(1):14-20. DOI: 10.1016/j.ijmyco.2015.10.002.
56. Ben Fredj N, Gam R, Kerkni E, Chaabane A, Chadly Z, Boughattas N, et al. Risk factors of isoniazid-induced hepatotoxicity in Tunisian tuberculosis patients. The pharmacogenomics journal 2016; 17(4):372-377. DOI: 10.1038/tpj.2016.26.
57. Bouazzi OE, Hammi S, Bourkadi JE, Tebaa A, Tanani DS, Soulaymani-Bencheikh R, et al. First line anti-tuberculosis induced hepatotoxicity: incidence and risk factors. The Pan African medical journal 2016; 25:167. DOI: 10.11604/pamj.2016.25.167.10060.
58. Carolline AM, Lopes EP, Acioli-Santos B, Maruza M, Montarroyos UR, Ximenes RA, et al. Hepatotoxicity during Treatment for Tuberculosis in People Living with HIV/AIDS. PloS one 2016; 11(6): e0157725. DOI: 10.1371/journal.pone.0157725.
59. Isa SE, Ebonyi AO, Shehu NY, Idoko P, Anejo-Okopi JA, Simji G, et al. Antituberculosis drugs and hepatotoxicity among hospitalized patients in Jos, Nigeria. International journal of mycobacteriology 2016; 5(1):21-26. DOI: 10.1016/j.ijmyco.2015.10.001.
60. Kim WS, Lee SS, Lee CM, Kim HJ, Ha CY, Kim HJ, et al. Hepatitis C and not Hepatitis B virus is a risk factor for anti-tuberculosis drug induced liver injury. BMC infectious diseases 2016; 16:50. DOI: 10.1186/s12879-016-1344-2.
61. Perwitasari DA, IHRam LM, Darmawan E, Mulyani UA, Atthobari J. CYP2E1 polymorphism, acetylator profiles and drug-induced liver injury incidence of Indonesian tuberculosis patients. The Indian journal of tuberculosis 2016; 63(3):139-143. DOI: 10.1016/j.ijtb.2016.08.001.
62. Saha A, Shanthi FXM, Winston AB, Das S, Kumar A, Michael JS, et al. Prevalence of Hepatotoxicity From Antituberculosis Therapy: A Five-Year Experience From South India. Journal of primary care & community health 2016; 7(3):171-174. DOI: 10.1177/2150131916642431.
63. Sun Q, Zhang Q, Gu J, Sun WW, Wang P, Bai C, et al. Prevalence, risk factors, management, and treatment outcomes of first-line antituberculous drug-induced liver injury: a prospective cohort study. Pharmacoepidemiology and drug safety 2016; 25(8):908-917. DOI: 10.1002/pds.3988.
64. Bright-Thomas RJ, Gondker AR, Morris J, Ormerod LP. Drug-related hepatitis in patients treated with standard antituberculosis chemotherapy over a 30-year period. International Journal of Tuberculosis and Lung Disease 2016; 20(12):1621-1624. DOI: 10.5588/ijtld.16.0370.
65. Vijayalakshmi A, Thanmayi G, Jayakumari S. A prospective study on abnormal liver function test patterns in patients receiving anti-tuberculosis therapy. Asian Journal of Pharmaceutical and Clinical Research 2016; 9(5):136-139. DOI: 10.22159/ajpcr.2016.v9i5.12756.
66. Wu S, Wang YJ, Tang X, Wang Y, Wu J, Ji G, et al. Genetic Polymorphisms of Glutathione S-Transferase P1 (GSTP1) and the Incidence of Anti-Tuberculosis Drug-Induced Hepatotoxicity. PloS one 2016; 11(6): e0157478. DOI: 10.1371/journal.pone.0157478.
67. Costiniuk CT, Gosnell BI, Manzini TC, Du Plessis CN, Moosa MYS. Tuberculous Drug-induced Liver Injury and Treatment Re-challenge in Human Immunodeficiency Virus Co-infection. Journal of global infectious diseases 2015; 7(4):151-156. DOI: 10.4103/0974-777x.170499.
68. Erin E Bliven-Sizemore, Sterling TR, Shang N, Benator D, Schwartzman K, Reves R, et al. Three months of weekly rifapentine plus isoniazid is less hepatotoxic than nine months of daily isoniazid for LTBI. Int J Tuberc Lung Dis 2015; 19(9):1039-1044. DOI: 10.5588/ijtld.14.0829.
69. Fernandes DC, Santos NP, Moraes MR, Braga AC, Silva CA, Ribeiro-dos-Santos A, et al. Association of the CYP2B6 gene with anti-tuberculosis drug-induced hepatotoxicity in a Brazilian Amazon population. International journal of infectious diseases: IJID : official publication of the International Society for Infectious Diseases 2015; 33:28-31. DOI: 10.1016/j.ijid.2014.04.011.
70. Gaude GS, Chaudhury A, Hattiholi J. Drug-induced hepatitis and the risk factors for liver injury in pulmonary tuberculosis patients. Journal of family medicine and primary care 2015; 4(2):238-243. DOI: 10.4103/2249-4863.154661.
71. Horita N, Miyazawa N, Yoshiyama T, Kojima R, Ishigatsubo Y, Kaneko T. Currently Used Low-Dose Pyrazinamide Does Not Increase Liver-Injury in the First Two Months of Tuberculosis Treatment. Internal medicine (Tokyo, Japan) 2015; 54(18):2315-2320. DOI: 10.2169/internalmedicine.54.5533.
72. Nooredinvand HA, Connell DW, Asgheddi M, Abdullah M, O'Donoghue M, Campbell L, et al. Viral hepatitis prevalence in patients with active and latent tuberculosis. World Journal of Gastroenterology 2015; 21(29):8974-8980. DOI: 10.3748/wjg.v21.i29.8920.
73. Sharma P, Tyagi P, Singla V, Bansal N, Kumar A, Arora A. Clinical and biochemical profile of tuberculosis in patients with liver cirHRosis. Journal of clinical and experimental hepatology 2015; 5(1):8-13. DOI: 10.1016/j.jceh.2015.01.003.
74. Tomich LGMM, Núñez M, Mendes-Correa MC. Drug-induced liver injury in hospitalized HIV patients: High incidence and association with drugs for tuberculosis. Annals of Hepatology 2015; 14(6):888-894. DOI: 10.5604/16652681.1171778.
75. Wang JY, Tsai CH, Lee YL, Lee LN, Hsu CL, Chang HC, et al. Gender-Dimorphic Impact of PXR Genotype and Haplotype on Hepatotoxicity During Antituberculosis Treatment. Medicine (Baltimore) 2015; 94(24):e982. DOI: 10.1097/md.0000000000000982.
76. Zazuli Z, Barliana MI, Mulyani UA, Perwitasari DA, Ng H, Abdulah R. Polymorphism of PXR gene associated with the increased risk of drug-induced liver injury in Indonesian pulmonary tuberculosis patients. 2015; 40(6):680-684. DOI: 10.1111/jcpt.12325.
77. Zhang T, Du J, Yin X, Xue F, Liu Y, Li R, et al. Adverse Events in Treating Smear-Positive Tuberculosis Patients in China. International journal of environmental research and public health 2015; 13(1):86; doi: 10.3390/ijerph13010086. DOI: 10.3390/ijerph13010086.
78. Abbasi MA, Ahmed N, Suleman A, Zaman H, Tariq S, Anwar SA, et al. Common risk factors for the development of anti-tuberculosis treatment induced hepatotoxicity. Journal of Ayub Medical College, Abbottabad: JAMC 2014; 26(3):384-388.
79. Agha MA, El-Mahalawy II, Seleem HM, Helwa MA. Prevalence of hepatitis C virus in patients with tuberculosis and its impact in the incidence of anti-tuberculosis drugs induced hepatotoxicity. Egyptian Journal of Chest Diseases and Tuberculosis 2014; 64(1):91-96. DOI: 10.1016/j.ejcdt.2014.09.009.
80. Castro ATE, Mendes M, Freitas S, Roxo PC. Incidence and risk factors of major toxicity associated to first-line antituberculosis drugs for latent and active tuberculosis during a period of 10 years. Revista Portuguesa de Pneumologia 2014; 21(3):144-150. DOI: 10.1016/j.rppnen.2014.08.004.
81. Chang SH, Nahid P, Eitzman SR. Hepatotoxicity in Children Receiving Isoniazid Therapy for Latent Tuberculosis Infection. Journal of the Pediatric Infectious Diseases Society 2014; 3(3):221-227. DOI: 10.1093/jpids/pit089.
82. Hatamkhani S, Khalili H, Karimzadeh I, Dashti-Khavidaki S, Abdollahi A, Jafari S. Carnitine for prevention of antituberculosis drug-induced hepatotoxicity: a randomized, clinical trial. Journal of gastroenterology and hepatology 2014; 29(5):997-1004. DOI: 10.1111/jgh.12474.
83. Liu YM, Cheng YJ, Li YL, Liu CE, Hsu WH. Antituberculosis treatment and hepatotoxicity in patients with chronic viral hepatitis. Lung 2014; 192(1):205-210. DOI: 10.1007/s00408-013-9535-8.
84. Mo P, Zhu Q, Teter C, Yang R, Deng L, Yan Y, et al. Prevalence, drug-induced hepatotoxicity, and mortality among patients multi-infected with HIV, tuberculosis, and hepatitis virus. International Journal of Infectious Diseases 2014; 28:e95-e100. DOI: 10.1016/j.ijid.2014.06.020.
85. Rana SV, Sharma SK, Ola RP, Kamboj JK, Malik A, Morya RK, et al. N-acetyltransferase 2, cytochrome P4502E1 and glutathione S-transferase genotypes in antitubercular treatment-induced hepatotoxicity in North Indians. Journal of clinical pharmacy and therapeutics 2014; 39(1):91-96. DOI: 10.1111/jcpt.12105.
86. Schultz V, Marroni CA, Amorim CS, Baethgen LF, Pasqualotto AC. Risk factors for hepatotoxicity in solid organ transplants recipients being treated for tuberculosis. Transplantation proceedings 2014; 46(10):3606-3610. DOI: 10.1016/j.transproceed.2014.09.148.
87. Xiang Y, Ma L, Wu W, Liu W, Li Y, Zhu X, et al. The incidence of liver injury in Uyghur patients treated for TB in Xinjiang Uyghur autonomous region, China, and its association with hepatic enzyme polymorphisms nat2, cyp2e1, gstm1 and gstt1. PloS one 2014; 9(1):e85905. DOI: 10.1371/journal.pone.0085905.
88. Zaverucha-do-Valle C, Monteiro SP, El-Jaick KB, Rosadas LA, Costa MJM, Quintana MSB, et al. The role of cigarette smoking and liver enzymes polymorphisms in anti-tuberculosis drug-induced hepatotoxicity in Brazilian patients. Tuberculosis (Edinburgh, Scotland) 2014; 94(3):299-305. DOI: 10.1016/j.tube.2014.03.006.
89. Zuberi BF, Zuberi FF, Bader N, Alvi H, Salahuddin J. Comparison of British Thoracic Society and American Thoracic Society reintroduction guidelines for anti-tuberculous therapy induced liver injury. JPMA The Journal of the Pakistan Medical Association 2014; 64(8):896-899.
90. Gupta VH, Amarapurkar DN, Singh M, Sasi P, Joshi JM, Baijal R, et al. Association of N-acetyltransferase 2 and cytochrome P450 2E1 gene polymorphisms with antituberculosis drug-induced hepatotoxicity in Western India. Journal of gastroenterology and hepatology 2013; 28(8):1368-1374. DOI: 10.1111/jgh.12194.
91. Hassen Ali A, Belachew T, Yami A, Ayen WY. Anti-tuberculosis drug induced hepatotoxicity among TB/HIV co-infected patients at Jimma University Hospital, Ethiopia: nested case-control study. PloS one 2013; 8(5):e64622. DOI: 10.1371/journal.pone.0064622.
92. Lomtadze N, Kupreishvili L, Salakaia A, Vashakidze S, Sharvadze L, Kempker RR, et al. Hepatitis C virus co-infection increases the risk of anti-tuberculosis drug-induced hepatotoxicity among patients with pulmonary tuberculosis. PloS one 2013; 8(12): e83892. DOI: 10.1371/journal.pone.0083892.
93. Santos NP, Callegari-Jacques SM, Ribeiro Dos Santos AK, Silva CA, Vallinoto AC, Fernandes DC, et al. N-acetyl transferase 2 and cytochrome P450 2E1 genes and isoniazid-induced hepatotoxicity in Brazilian patients. Int J Tuberc Lung Dis 2013; 17(4):499-504. DOI: 10.5588/ijtld.12.0645.
94. Shu CC, Lee CH, Lee MC, Wang JY, Yu CJ, Lee LN. Hepatotoxicity due to first-line anti-tuberculosis drugs: a five-year experience in a Taiwan medical centre. The international journal of tuberculosis and lung disease: the official journal of the International Union against Tuberculosis and Lung Disease 2013; 17(7):934-939. DOI: 10.5588/ijtld.12.0782.
95. Babalik A, Arda H, Bakirci N, Aǧca S, Oruç K, Kiziltaş Ş, et al. Management of and risk factors related to hepatotoxicity during tuberculosis treatment. Tuberkuloz ve Toraks 2012; 60(2):136-144. DOI: 10.5578/tt.3053.
96. Keshavjee S, Gelmanova IY, Shin SS, Mishustin SP, Andreev YG, Atwood S, et al. Hepatotoxicity during treatment for multidrug-resistant tuberculosis: occurrence, management and outcome. The international journal of tuberculosis and lung disease: the official journal of the International Union against Tuberculosis and Lung Disease 2012; 16(5):596-603. DOI: 10.5588/ijtld.11.0591.
97. Mansukhani S, Shah I. Hepatic dysfunction in children with tuberculosis on treatment with antituberculous therapy. Annals of Hepatology 2012; 11(1):96-99.
98. Monteiro TP, El-Jaick KB, Jeovanio-Silva AL, Brasil PEAA, Costa MJM, Rolla VC, et al. The roles of GSTM1 and GSTT1 null genotypes and other predictors in anti-tuberculosis drug-induced liver injury. Journal of clinical pharmacy and therapeutics 2012; 37(6):712-718. DOI: 10.1111/j.1365-2710.2012.01368.x.
99. Nanashima K, Mawatari T, Tahara N, Higuchi N, Nakaura A, Inamine T, et al. Genetic variants in antioxidant pathway: risk factors for hepatotoxicity in tuberculosis patients. Tuberculosis (Edinburgh, Scotland) 2012; 92(3):253-259. DOI: 10.1016/j.tube.2011.12.004.
100. Singanayagam A, Sridhar S, Dhariwal J, Abdel-Aziz D, Munro K, Connell DW, et al. A comparison between two strategies for monitoring hepatic function during antituberculous therapy. American Journal of Respiratory and Critical Care Medicine 2012; 185(6):653-659. DOI: 10.1164/rccm.201105-0850OC.
101. Thongraung W, Sittidach M, Khwansuwan P, Sariyasuntorn K, Wongsampan S. Evaluation of the physicians' approach to the diagnosis and treatment of patients with antituberculosis drug-induced hepatotoxicity. Journal of evaluation in clinical practice 2012; 18(6):1119-1125. DOI: 10.1111/j.1365-2753.2011.01706.x.
102. Bose PD, Sarma MP, Medhi S, Das BC, Husain SA, Kar P. Role of polymorphic N-acetyl transferase2 and cytochrome P4502E1 gene in antituberculosis treatment-induced hepatitis. Journal of gastroenterology and hepatology 2011; 26(2):312-318. DOI: 10.1111/j.1440-1746.2010.06355.x.
103. Leiro-Fernandez V, Valverde D, Vázquez-Gallardo R, Botana-Rial M, Constenla L, Agúndez JA, et al. N-acetyltransferase 2 polymorphisms and risk of anti-tuberculosis drug-induced hepatotoxicity in Caucasians. 2011; 15(10):1403-1408. DOI: 10.5588/ijtld.10.0648.
104. Lorent N, Sebatunzi O, Mukeshimana G, Van den Ende J, Clerinx J. Incidence and risk factors of serious adverse events during antituberculous treatment in Rwanda: a prospective cohort study. PloS one 2011; 6(5):e19566. DOI: 10.1371/journal.pone.0019566.
105. Mankhatitham W, Lueangniyomkul A, Manosuthi W. Hepatotoxicity in patients co-infected with tuberculosis and HIV-1 while receiving non-nucleoside reverse transcriptase inhibitor-based antiretroviral therapy and rifampicin-containing anti-tuberculosis regimen. The Southeast Asian journal of tropical medicine and public health 2011; 42(3):651-658.
106. Shang P, Xia Y, Liu F, Wang X, Yuan Y, Hu D, et al. Incidence, clinical features and impact on anti-tuberculosis treatment of anti-tuberculosis drug induced liver injury (ATLI) in China. PloS one 2011; 6(7):e21836. DOI: 10.1371/journal.pone.0021836.
107. Teixeira RLDF, Morato RG, Cabello PH, Muniz LMK, Moreira ASR, Kritski AL, et al. Genetic polymorphisms of NAT2, CYP2E1 and GST enzymes and the occurrence of antituberculosis drug-induced hepatitis in Brazilian TB patients. Memorias do Instituto Oswaldo Cruz 2011; 106(6):716-724. DOI: 10.1590/S0074-02762011000600011.
108. Baghaei P, Tabarsi P, Chitsaz E, Saleh M, Marjani M, Shemirani S, et al. Incidence, clinical and epidemiological risk factors, and outcome of drug-induced hepatitis due to antituberculous agents in new tuberculosis cases. American journal of therapeutics 2010; 17(1):17-22. DOI: 10.1097/MJT.0b013e31818f9eae.
109. Coca NSM, Oliveira MS, Voieta I, Antunes CMF, Lambertucci JR. Antituberculosis drug-induced hepatotoxicity: A comparison between patients with and without human immunodeficiency virus seropositivity. Revista da Sociedade Brasileira de Medicina Tropical 2010; 43(6):624-628.
110. [86] Khoharo HK, Ansari S, Siddiqui AA, Qureshi F. Standard antituberculosis drug induced hepatotoxicity: Do the risk factors matter? Journal of the Liaquat University of Medical and Health Sciences 2010; 9(2):84-87.
111. Lee SW, Chung LSC, Huang HH, Chuang TY, Liou YH, Wu LSH. NAT2 and CYP2E1 polymorphisms and susceptibility to fi rst-line anti-tuberculosis drug-induced hepatitis. International Journal of Tuberculosis and Lung Disease 2010; 14(5):622-626.
112. Moses M, Zachariah R, Tayler-Smith K, Misinde D, Foncha C, Manzi M, et al. Outcomes and safety of concomitant nevirapine and rifampicin treatment under programme conditions in Malawi. The international journal of tuberculosis and lung disease: the official journal of the International Union against Tuberculosis and Lung Disease 2010; 14(2):197-202.
113. Nader LA, de Mattos AA, Picon PD, Bassanesi SL, De Mattos AZ, Pineiro Rodriguez M. Hepatotoxicity due to rifampicin, isoniazid and pyrazinamide in patients with tuberculosis: is anti-HCV a risk factor? Annals of hepatology 2010; 9(1):70-74.
114. Naz S, Marwat JUD, Arshad M, Ahmed A, Khan HR, Ullah Z. Assessment of risk factors for anti-tuberculous drug induced hepatotoxicity. Journal of Medical Sciences 2010; 18(1):45-48.
115. Park WB, Kim W, Lee KL, Yim JJ, Kim M, Jung YJ, et al. Antituberculosis drug-induced liver injury in chronic hepatitis and cirHRosis. The Journal of infection 2010; 61(4):323-329. DOI: 10.1016/j.jinf.2010.07.009.
116. Sharma SK, Singla R, Sarda P, Mohan A, Makharia G, Jayaswal A, et al. Safety of 3 different reintroduction regimens of antituberculosis drugs after development of antituberculosis treatment-induced hepatotoxicity. Clinical Infectious Diseases 2010; 50(6):833-839. DOI: 10.1086/650576.
117. Tedla Z, Nyirenda S, Peeler C, Agizew T, Sibanda T, Motsamai O, et al. Isoniazid-associated hepatitis and antiretroviral drugs during tuberculosis prophylaxis in hiv-infected adults in Botswana. American journal of respiratory and critical care medicine 2010; 182(2):278-285. DOI: 10.1164/rccm.200911-1783OC.
118. Tostmann A, van den Boogaard J, Semvua H, Kisonga R, Kibiki GS, Aarnoutse RE, et al. Antituberculosis drug-induced hepatotoxicity is uncommon in Tanzanian hospitalized pulmonary TB patients. Tropical medicine & international health: TM & IH 2010; 15(2):268-272. DOI: 10.1111/j.1365-3156.2009.02449.x.
119. Fountain FF, Tolley EA, Jacobs AR, Self TH. Rifampin hepatotoxicity associated with treatment of latent tuberculosis infection. The American journal of the medical sciences 2009; 337(5):317-320. DOI: 10.1097/MAJ.0b013e31818c0134.
120. Walker NF, Kliner M, Turner D, Bhagani S, Cropley I, Hopkins S, et al. Hepatotoxicity and antituberculosis therapy: time to revise UK guidance? Thorax 2009; 64(10):918. DOI: 10.1136/thx.2009.115469.
121. Kim SH, Kim SH, Bahn JW, Kim YK, Chang YS, Shin ES, et al. Genetic polymorphisms of drug-metabolizing enzymes and anti-TB drug-induced hepatitis. Pharmacogenomics 2009; 10(11):1767-1779. DOI: 10.2217/pgs.09.100.
122. Chang KC, Leung CC, Yew WW, Lau TY, Tam CM. Hepatotoxicity of pyrazinamide: cohort and case-control analyses. American journal of respiratory and critical care medicine 2008; 177(12):1391-1396. DOI: 10.1164/rccm.200802-355OC.
123. Makhlouf HA, Helmy A, Fawzy E, El-Attar M, Rashed HAG. A prospective study of antituberculous drug-induced hepatotoxicity in an area endemic for liver diseases. Hepatology International 2008; 2(3):353-360. DOI: 10.1007/s12072-008-9085-y.
124. Marzuki OA, Fauzi ARM, Ayoub S, Kamarul Imran M. Prevalence and risk factors of anti-tuberculosis drug-induced hepatitis in Malaysia. Singapore Medical Journal 2008; 49(9):688-693.
125. Possuelo LG, Castelan JA, De Brito TC, Ribeiro AW, Cafrune PI, Picon PD, et al. Association of slow N-acetyltransferase 2 profile and anti-TB drug-induced hepatotoxicity in patients from Southern Brazil. European Journal of Clinical Pharmacology 2008; 64(7):673-681. DOI: 10.1007/s00228-008-0484-8.
126. Yimer G, Aderaye G, Amogne W, Makonnen E, Aklillu E, Lindquist L, et al. Anti-tuberculosis therapy-induced hepatotoxicity among Ethiopian HIV-positive and negative patients. PloS one 2008; 3(3):e1809. DOI: 10.1371/journal.pone.0001809.
127. Cho HJ, Koh WJ, Ryu YJ, Ki CS, Nam MH, Kim JW, et al. Genetic polymorphisms of NAT2 and CYP2E1 associated with antituberculosis drug-induced hepatotoxicity in Korean patients with pulmonary tuberculosis. 2007; 87(6):551-556. DOI: 10.1016/j.tube.2007.05.012.
128. Kwon YS, Koh WJ, Suh GY, Chung MP, Kim H, Kwon OJ. Hepatitis C virus infection and hepatotoxicity during antituberculosis chemotherapy. Chest 2007; 131(3):803-808. DOI: 10.1378/chest.06-2042.
129. Mahmood K, Hussain A, Jairamani KL, Talib A, Abbasi BU, Salkeen S. Hepatotoxicity with antituberculosis drugs: The risk factors. Pakistan Journal of Medical Sciences 2007; 23(1):33-38.
130. Pukenyte E, Lescure FX, Rey D, Rabaud C, Hoen B, Chavanet P, et al. Incidence of and risk factors for severe liver toxicity in HIV-infected patients on anti-tuberculosis treatment. The international journal of tuberculosis and lung disease: the official journal of the International Union against Tuberculosis and Lung Disease 2007; 11(1):78-84.
131. Cook PP, Maldonado RA, Yarnell CT, Holbert D. Safety and completion rate of short-course therapy for treatment of latent tuberculosis infection. Clinical infectious diseases: an official publication of the Infectious Diseases Society of America 2006; 43(3):271-275. DOI: 10.1086/505398.
132. Gülbay BE, Gürkan OU, Yildiz OA, Onen ZP, Erkekol FO, Baççioğlu A, et al. Side effects due to primary antituberculosis drugs during the initial phase of therapy in 1149 hospitalized patients for tuberculosis. Respiratory medicine 2006; 100(10):1834-1842. DOI: 10.1016/j.rmed.2006.01.014.
133. Senaratne WV, Pinidiyapathirage MJ, Perera GA, Wickremasinghe AR. Anti-tuberculosis drug inducd hepatitis - a Sri Lankan experience. The Ceylon medical journal 2006; 51(1):9-14. DOI: 10.4038/cmj.v51i1.1369.
134. Vuilleumier N, Rossier MF, Chiappe A, Degoumois F, Dayer P, Mermillod B, et al. CYP2E1 genotype and isoniazid-induced hepatotoxicity in patients treated for latent tuberculosis. European journal of clinical pharmacology 2006; 62(6):423-429. DOI: 10.1007/s00228-006-0111-5.
135. Fountain FF, Tolley E, Chrisman CR, Self TH. Isoniazid hepatotoxicity associated with treatment of latent tuberculosis infection: a 7-year evaluation from a public health tuberculosis clinic. Chest 2005; 128(1):116-123. DOI: 10.1378/chest.128.1.116.
136. Meier Y, Cavallaro M, Roos M, Pauli-Magnus C, Folkers G, Meier PJ, et al. Incidence of drug-induced liver injury in medical inpatients. European journal of clinical pharmacology 2005; 61(2):135-143. DOI: 10.1007/s00228-004-0888-z.
137. Shakya R, Rao BS, Shrestha B. Management of antitubercular drugs-induced hepatotoxicity and therapy reintroduction strategy in a TB clinic of Nepal. Kathmandu University medical journal (KUMJ) 2005; 3(1):45-49.
138. Sharifzadeh M, Rasoulinejad M, Valipour F, Nouraie M, Vaziri S. Evaluation of patient-related factors associated with causality, preventability, predictability and severity of hepatotoxicity during antituberclosis treatment. Pharmacological Research 2005; 51(4):353-358. DOI: 10.1016/j.phrs.2004.10.009.
139. Kandula NR, Dworkin MS, Carroll MR, Lauderdale DS. Tuberculosis prevention in Mexican immigrants: limitations of short-course therapy. American journal of preventive medicine 2004; 26(2):163-166. DOI: 10.1016/j.amepre.2003.10.011.
140. Menzies D, Dion MJ, Rabinovitch B, Mannix S, Brassard P, Schwartzman K. Treatment completion and costs of a randomized trial of rifampin for 4 months versus isoniazid for 9 months. American Journal of Respiratory and Critical Care Medicine 2004; 170(4):445-449. DOI: 10.1164/rccm.200404-478OC.
141. Pedral-Sampaio DB, Alves CR, Netto EM, Brites C, Oliveira AS, Badaro R. Efficacy and safety of Efavirenz in HIV patients on Rifampin for tuberculosis. The Brazilian journal of infectious diseases: an official publication of the Brazilian Society of Infectious Diseases 2004; 8(3):211-216. DOI: 10.1590/s1413-86702004000300004.
142. Huang YS, Chern HD, Su WJ, Wu JC, Chang SC, Chiang CH, et al. Cytochrome P450 2E1 genotype and the susceptibility to antituberculosis drug-induced hepatitis. Hepatology (Baltimore, Md) 2003; 37(4):924-930. DOI: 10.1053/jhep.2003.50144.
143. LoBue PA, Moser KS. Use of isoniazid for latent tuberculosis infection in a public health clinic. American journal of respiratory and critical care medicine 2003; 168(4):443-447.DOI: 10.1164/rccm.200303-390OC.
144. Stout JE, Engemann JJ, Cheng AC, Fortenberry ER, Hamilton CD. Safety of 2 months of rifampin and pyrazinamide for treatment of latent tuberculosis. American journal of respiratory and critical care medicine 2003; 167(6):824-827. DOI: 10.1164/rccm.200209-998OC.
145. Yee D, Valiquette C, Pelletier M, Parisien I, Rocher I, Menzies D. Incidence of serious side effects from first-line antituberculosis drugs among patients treated for active tuberculosis. American journal of respiratory and critical care medicine 2003; 167(11):1472-1477. DOI: 10.1164/rccm.200206-626OC.
146. Jasmer R, Saukkonen J, Blumberg H, Daley C, Bernardo J, Vittinghoff E, et al. Short-course rifampin and pyrazinamide compared with isoniazid for latent tuberculosis infection: a multicenter clinical trial. In: Annals of internal medicine; 2002.137:640-647. DOI: 10.7326/0003-4819-137-8-200210150-00007.
147. Lee AM, Mennone JZ, Jones RC, Paul WS. Risk factors for hepatotoxicity associated with rifampin and pyrazinamide for the treatment of latent tuberculosis infection: experience from three public health tuberculosis clinics. The international journal of tuberculosis and lung disease: the official journal of the International Union against Tuberculosis and Lung Disease 2002; 6(11):995-1000.
148. Ohkawa K, Hashiguchi M, Ohno K, Kiuchi C, Takahashi S, Kondo S, et al. Risk factors for antituberculous chemotherapy-induced hepatotoxicity in Japanese pediatric patients. Clinical pharmacology and therapeutics 2002; 72(2):220-226. DOI: 10.1067/mcp.2002.126175.
149. Teleman MD, Chee CBE, Earnest A, Wang YT. Hepatotoxicity of tuberculosis chemotherapy under general programme conditions in Singapore. The international journal of tuberculosis and lung disease: the official journal of the International Union against Tuberculosis and Lung Disease 2002; 6(8):699-705.
150. Ohno M, Yamaguchi I, Yamamoto I, Fukuda T, Yokota S, Maekura R, et al. Slow N-acetyltransferase 2 genotype affects the incidence of isoniazid and rifampicin-induced hepatotoxicity. The international journal of tuberculosis and lung disease: the official journal of the International Union against Tuberculosis and Lung Disease 2000; 4(3):256-261.
151. Ungo JR, Jones D, Ashkin D, Hollender ES, Bernstein D, Albanese AP, et al. Antituberculosis drug-induced hepatotoxicity. The role of hepatitis C virus and the human immunodeficiency virus. American journal of respiratory and critical care medicine 1998; 157(6 Pt 1):1871-1876. DOI: 10.1164/ajrccm.157.6.9711039.
152. Hwang SJ, Wu JC, Lee CN, Yen FS, Lu CL, Lin TP, et al. A prospective clinical study of isoniazid-rifampicin-pyrazinamide-induced liver injury in an area endemic for hepatitis B. Journal of gastroenterology and hepatology 1997; 12(1):87-91. DOI: 10.1111/j.1440-1746.1997.tb00353.x.
153. Døssing M, Wilcke JT, Askgaard DS, Nybo B. Liver injury during antituberculosis treatment: an 11-year study. Tubercle and lung disease: the official journal of the International Union against Tuberculosis and Lung Disease 1996; 77(4):335-340. DOI: 10.1016/s0962-8479(96)90098-2.
154. Ozick LA, Jacob L, Comer GM, Lee TP, Ben-Zvi J, Donelson SS, et al. Hepatotoxicity from isoniazid and rifampin in inner-city AIDS patients. The American journal of gastroenterology 1995; 90(11):1978-1980.
155. Barazzone C, Hofer M, Nussle D, Suter S, Rochat T. Childhood tuberculosis at a Swiss university hospital: a 2-year study. European journal of pediatrics 1993; 152(10):805-809. DOI: 10.1007/bf02073375.
156. Aziz S, Agha F, Hassan R, Fairoz SA, Hassan K. Hepatotoxicity to different antituberculosis drug combinations. JPMA The Journal of the Pakistan Medical Association 1990; 40(12):290-294.
